# Lichen-like association of *Chlamydomonas reinhardtii* and *Aspergillus nidulans* protects algal cells from bacteria

**DOI:** 10.1038/s41396-020-0731-2

**Published:** 2020-08-04

**Authors:** Mario K. C. Krespach, María García-Altares, Michal Flak, Hanno Schoeler, Kirstin Scherlach, Tina Netzker, Anica Schmalzl, Derek J. Mattern, Volker Schroeckh, Anna Komor, Maria Mittag, Christian Hertweck, Axel A. Brakhage

**Affiliations:** 1 Department of Molecular and Applied Microbiology, Leibniz Institute for Natural Product Research and Infection Biology (HKI), Jena, Germany; 2 Institute for Microbiology, Friedrich Schiller University Jena, Jena, Germany; 3 Department of Biomolecular Chemistry, Leibniz Institute for Natural Product Research and Infection Biology (HKI), Jena, Germany; 4 Matthias Schleiden Institute of Genetics, Bioinformatics, and Molecular Botany, Friedrich Schiller University Jena, Jena, Germany; 5 Present Address: Metabolomics Platform, Department of Electronic Engineering (DEEEA), Universitat Rovira i Virgili, Tarragona, Spain; 6 Present Address: Biologie des Bactéries Intracellulaires, Institut Pasteur, 28 rue du Dr. Roux, 75015 Paris, France; 7 Present Address: Department of Biology, McMaster University, 1280 Main Street West, Hamilton, ON L8S 4K1 Canada

**Keywords:** Microbial ecology, Microbiome, Microbial ecology, Antibiotics, Fungal ecology

## Abstract

Organismal interactions within microbial consortia and their responses to harmful intruders remain largely understudied. An important step toward the goal of understanding functional ecological interactions and their evolutionary selection is the study of increasingly complex microbial interaction systems. Here, we discovered a tripartite biosystem consisting of the fungus *Aspergillus nidulans*, the unicellular green alga *Chlamydomonas reinhardtii*, and the algicidal bacterium *Streptomyces iranensis*. Genetic analyses and MALDI-IMS demonstrate that the bacterium secretes the algicidal compound azalomycin F upon contact with *C. reinhardtii*. In co-culture, *A. nidulans* attracts the motile alga *C. reinhardtii*, which becomes embedded and surrounded by fungal mycelium and is shielded from the algicide. The filamentous fungus *Sordaria macrospora* was susceptible to azalomycin F and failed to protect *C. reinhardtii* despite chemotactically attracting the alga. Because *S. macrospora* was susceptible to azalomycin F, this data imply that for protection the fungus needs to be resistant. Formation of the lichen-like association between *C. reinhardtii* and *A. nidulans* increased algal growth. The protection depends on the increased amounts of membrane lipids provided by resistant fungi, thereby generating a protective shelter against the bacterial toxin. Our findings reveal a strategy whereby algae survive lethal environmental algicides through cooperation with fungi.

## Introduction

Algae and fungi have coexisted and coevolved for at least 400 million years, as deduced from fossil records showing macroalgae parasitized by fungi [1], as well as lichens [2, 3]. During the course of evolution, terrestrial algae and fungi have formed lichens but some have lost this ability [4]. Tight associations between algae and fungi, in particular in lichens, provide examples of mutualism that has been maintained over hundreds of millions of years up to the present [4]. However, the benefits for the algal partners remain a matter of debate as the mycobiont often gains a fitness advantage at the expense of the photobiont. Furthermore, the mycobiont can control the growth and metabolism of the photobiont, which is referred to as “helotism” [5], a type of “algal slavery” [6] with reference to the master–servant relationship of ancient sparta.

Microorganisms are often subject to rapidly changing biotic and abiotic conditions in their natural habitats. To cope with these environmental changes, they often produce natural products that are low molecular mass metabolites [7, 8]. They are often dispensable for growth, produced at a certain stage in the life cycle of the producing organism and can be of pharmaceutical value [7]. In many cases, these natural products are weapons to defend habitats against competing microorganisms. Metagenomic analysis has revealed that lichens are colonized by various other microorganisms and form “microhabitats” themselves [9, 10]. Among the potentially beneficial colonizers, there are streptomycetes equipped with an antibiotic arsenal [11–14]. How this multipartner interaction remains stable through mutualistic exchange and how lichens cope with potentially toxin-producing colonizers is largely unknown.

Co-existance of filamentous fungi, streptomycetes and other producers of natural products often triggers the production of these effector chemicals [15]. For instance, *Streptomyces rapamycinicus* and its closest relative *S. iranensis* (Supplementary Fig. 1) are capable of eliciting the production of the archetypical lichen metabolites orsellinic acid and lecanoric acid in the fungal partner *A. nidulans*. However these metabolites do not show activity against *S. rapamycinicus*, the closest relative to *S. iranensis* [16–18]. Since *A. nidulans* interacts with the green alga *C. reinhardtii* [19] and lichens are colonized by potentially harmful streptomycetes [11, 12, 14], we hypothesized that *S. iranensis* might interfere with this algal–fungal association. Here, we have found a tripartite interaction of the model green alga *C. reinhardtii* with the established bipartite *A. nidulans-S. iranensis* co-culture system [17]. We found that both partners have distinct effects on the alga: (i) the bacterium kills the alga using the natural product azalomycin F, (ii) the alga is protected by fungi resistant against azalomycin F like *A. nidulans*, and (iii) the presence of *A. nidulans* increases the growth rate and overall biomass of *C. reinhardtii*. We show that the algal–fungal association itself is a defense mechanism against toxins.

## Materials and methods

### Microbial strains and plasmids

Microbial strains and plasmids used in this study are listed in Supplementary Table 1.

### *Streptomyces iranensis* mutants

For deleting genes of *S. iranensis* the method described by Netzker et al. [20] was essentially followed. Oligonucleotides applied for gene deletion are listed in Supplementary Table 2.

### Production and purification of azalomycin F from *S. iranensis*

To obtain samples of the azalomycin F complex from *S. iranensis*, 50 mL of TSBY [21] were inoculated with 5 × 10^8^ spores and the bacteria were incubated for 4 days at 28 °C at 180 rpm. The entire culture was centrifuged to separate the biomass from the culture supernatant. After lyophilisation of the biomass, the content of the pellet was extracted twice with 50 mL methanol at 60 °C. Both extraction solutions were combined and evaporated to dryness under reduced pressure and dissolved in 2 mL 75% (v/v) aqueous methanol (MeOH_(aq)_). The extract was subsequently loaded onto an N-vinylpyrrolidone-divinylbenzene copolymer resin SPE column (Macherey-Nagel, Düren, Germany), washed with 50% (v/v) MeOH_(aq)_ and eluted with 70% (v/v) MeOH_(aq)_ to obtain purified azalomycin F complex. The eluent was reduced to dryness, dissolved in pure MeOH, and 5 μL were loaded onto an ultrahigh-performance liquid chromatography-mass spectrometry for analysis. The chromatographic system consisted of an UltiMate 3000 binary rapid-separation liquid chromatograph with photodiode array detector (Thermo Fisher Scientific, Dreieich, Germany). The MS was performed on an LTQ XL linear ion trap mass spectrometer (Thermo Fisher Scientific, Dreieich, Germany) equipped with an electrospray ion source. The sample was analyzed on a 150 mm by 4.6 mm Accucore reversed-phase (RP)-MS column with a particle size of 2.6 μm (Thermo Fisher Scientific, Dreieich, Germany) at a flow rate of 1 mL/min with the following gradient: 0.1% (v/v) HCOOH-MeCN/0.1% (v/v) HCOOH–H_2_O 0/100, increased to 80/20 in 15 min and then to 100/0 in the following 20 min, held at 100/0 for 2 min, and reversed to 0/100 in 2 min, with detection range at 190–400 nm. For the azalomycin F-deficient mutants, cultivation, extraction, and analysis of azalomycin F are described in the Supplementary Methods Section 1.3.

### Sample preparation for matrix-assisted laser desorption ionization mass spectrometry imaging (MALDI-IMS)

MALDI-IMS was carried out on indium tin oxide (ITO) covered glass slides (Bruker Daltonics, Bremen, Germany). Slides were sterilized over a Bunsen burner and 3 mL of 1% (w/v) TAP [22] agar were spread onto the slide for *S. iranensis* monoculture. For co-cultivation, 1.5 mL 1% (w/v) TAP agar was spread onto one half of the ITO glass slide and allowed to solidify. The other half was covered with TAP agar, containing submerged *C. reinhardtii*. For this purpose, a *C. reinhardtii* culture was centrifuged at 3500 × *g* for 5 min and the supernatant was discarded. The pellet was resuspended in liquid TAP agar with a temperature of about 35 °C to yield an OD_750_ of 2. This suspension was then added to the other side of the slide and allowed to solidify. Finally, 15 µL *S. iranensis* mycelium, prewashed in PBS, was set between both agar sides. The slides were incubated at 26 °C and a light intensity of 30 µE m^−2^ s^−1^ in petri dishes for 5 days and subsequently dried in a hybridization oven at 37 °C for 48 h. For MALDI-IMS analysis, see Supplementary Methods 1.5.

### Chemotaxis assay

Chemotaxis was measured by counting *C. reinhardtii* cells that migrated into glass capillaries loaded with a potential attractant [23, 24]. Three microliters capillaries (Drummond Microcaps^®^, Drummond Scientific, Broomall, PA, USA) were loaded with TAP supplemented with 10 g/L glucose, 0.5 g/L MgCl_2_∙7H_2_O, 1 mL/L trace elements, 10 mL/L 0.5 M arginine, 3 mL/L 3 mM FeSO_4_ and 3 mL/L 0.1% (w/v) *p*-aminobenzoic acid (PABA) as control. Equally supplemented supernatant of an *A. nidulans* culture grown in TAP medium served as experimental sample and as a negative control PBS was used. The capillaries were placed upright into a 2 mL reaction tube. For each sample, three technical replicates were tested. 100 µL of a *C. reinhardtii* suspension with an OD_750_ of 4 were placed at the bottom of each 2 mL reaction tube. When attracted, the *C. reinhardtii* cells had to swim against gravity. Incubation was carried out at 26 °C in the dark for 5 h. After incubation, the capillaries were cleaned with a paper towel to remove attached cells, and the content was transferred to 27 µL of PBS + 4% (v/v) paraformaldehyde. *C. reinhardtii* cells which had migrated into the capillaries were counted using a Neubauer counting chamber.

### Chlorophyll quantification assay

The assay was performed in order to assess the chlorophyll content within a *C. reinhardtii* suspension and to estimate the viability of the cells 24 h after treatment with azalomycin F. Each well was inoculated with 100 µL TAP supplemented with glucose, MgSO_4_, PABA, FeSO_4_, trace elements, and arginine (see Supplementary Information 1.1) and containing 10^4^ conidia of *A. nidulans*. The 96-well plates were incubated at 37 °C for 16–18 h in a plate shaker with 1000 rpm. Then, 100 µL of a *C. reinhardtii* suspension with an OD_750_ of 2 was added to each well and fluorescence was measured (excitation 480 nm, emission 684 nm) on a Tecan Infinite M200 pro microplate reader (Tecan Trading AG, Männedorf, Switzerland). To allow *C. reinhardtii* to migrate into the mycelium, the plate was incubated for 5 h at 26 °C, 120 rpm and 30 µE m^−2^ s^−1^. Then, 200 ng azalomycin F were added and the fluorescence was measured again to determine the values for the start of the treatment. Incubation was continued for 24 h before measuring fluorescence using an excitation wavelength of 480 nm and measuring emission at 684 nm.

### Co-cultivation chamber experiments

Co-cultivation chambers were assembled as previously reported by Paul et al. [25]. The cultivation chambers were separated by a polyvinylidene fluoride (PVDF) membrane (Merck, Darmstadt, Germany) with a pore size of 0.1 µm. Each chamber was filled with 250 mL TAP supplemented with glucose, MgSO_4_, PABA, FeSO_4_, trace elements, and arginine (see Supplementary Information 1.1). *C. reinhardtii* was inoculated to obtain a final OD_750_ of 0.1 and *A. nidulans* mycelium was inoculated from a Miracloth-filtered overnight culture. The chambers were incubated at 26 °C, 80 rpm and 30 µE m^−2^ s^−1^. OD_750_ measurement was conducted on SERVA Nanophotometer Pearl (SERVA Heidelberg, Germany). After 24 h, 12.5 mL of 20% (w/v) glucose, 0.5 g/L MgSO_4_∙7H_2_O, 750 µL of 0.1% (w/v) PABA, 750 µL of 10 mM FeSO_4_, and 2.5 mL of 0.5 M L-arginine were added to each half of the co-cultivation chamber. *A. nidulans* dry mass was determined by filtering the mycelium after the experiment and drying it for 7 days at 60 °C.

### Protection of *C. reinhardtii* by external polar lipids

Phosphatidylinositol sodium salt from soybean was purchased from Sigma-Aldrich (St. Louis, MO, USA). Cardiolipin (CL), 1,2-dipalmitoyl-*sn*-glycero-3-phosphoglycerol (DPPG), 16:0-i15:0 phosphatidylcholine (PC), d18:1/18:0 C18 glucosyl(β)ceramide (GC), and extract of polar lipids of *Saccharomyces cerevisiae* (YE) were purchased from Avanti Polar Lipids (Alabaster, AL, USA). DPPG, CL, and GC were dissolved in 95:5 methanol:water to obtain a 0.5 mg/mL stock solution. PC was dissolved in 95:5 methanol:water to obtain a 4 mg/mL stock solution. YE was purchased as a 25 mg/mL solution in chloroform. For application of the lipids to *C. reinhardtii*, the solvent was evaporated under a gentle stream of nitrogen. The lipids were dissolved in a culture of *C. reinhardtii* with an OD_750_ of 2. The final concentrations of the lipids were chosen similar to Xu et al. [26]. To dissolve the lipids in TAP, the cultures were incubated in a ThermoMixer^®^ (Eppendorf, Hamburg, Germany) at 30 °C and rapid shaking for 30 min. Afterwards, the cultures were incubated in 24 well plates at 26 °C, 120 rpm and 30 µE m^−2^ s^−1^. Autofluorescence was measured after 24 h of incubation as described for the chlorophyll quantification assay.

## Results

### *Streptomyces iranensis* specifically releases algicidal azalomycin F in presence of *C. reinhardtii*

**Fig. 1:**
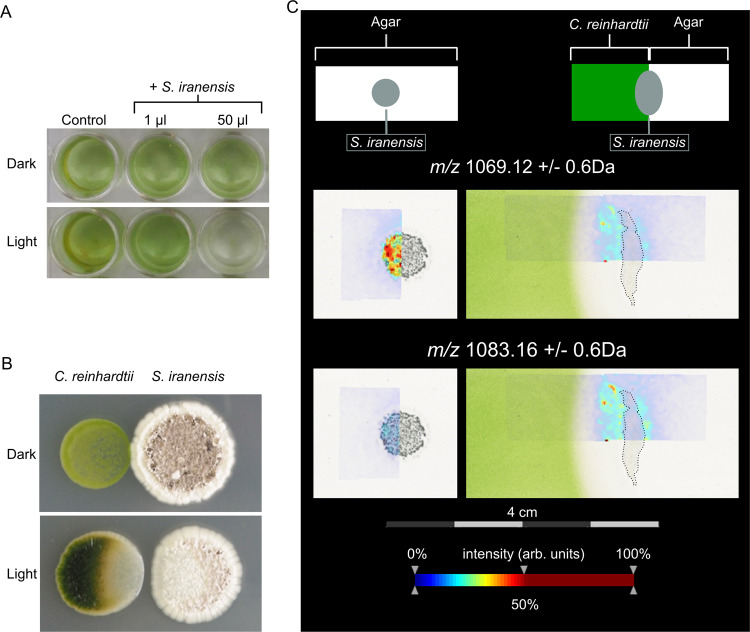
*S. iranensis* actively secretes azalomycin F showing light dependent algicidal activity. Co-cultures of *S. iranensis* and *C. reinhardtii* (**a**, **b**). Co-cultivation in light and dark with and without *S. iranensis* in TAP medium (**a**) and on agar (**b**). Loss of green coloration indicates killing of *C. reinhardtii*. **c** MALDI-IMS analysis. Top: schematic position of the *S. iranensis* colony, sterile TAP agar and TAP agar containing *C. reinhardtii* on the ITO-slide. MALDI-IMS images indicate color-coded abundance of ion *m/z* = 1069.12 ± 0.6 Da (azalomycin F3a, [M + H]^+^) and ion *m/z* = 1083.16 ± 0.6 Da (azalomycin F4a, [M + H]^+^). Color code: blue = low abundance, red = high abundance of ion. MALDI-IMS shows that both azalomycins are released only in co-cultivation of *S. iranensis* with *C. reinhardtii* which was inoculated into the left half of the agar.

*A. nidulans* and the green alga *C. reinhardtii* quickly assemble in co-culture to form an algal–fungal association [19]. We conducted co-cultivation experiments of *C. reinhardtii* and *S. iranensis* to identify possible effects of the bacterium on the alga. When co-cultured with *S. iranensis* under light, *C. reinhardtii* lost its green color in liquid medium (Fig. 1a) as well as on solid agar (Fig. 1b). Because decolorization is indicative of cell death of *C. reinhardtii* [27], *S. iranensis* likely produces one or several algicidal agents.

**Fig. 2:**
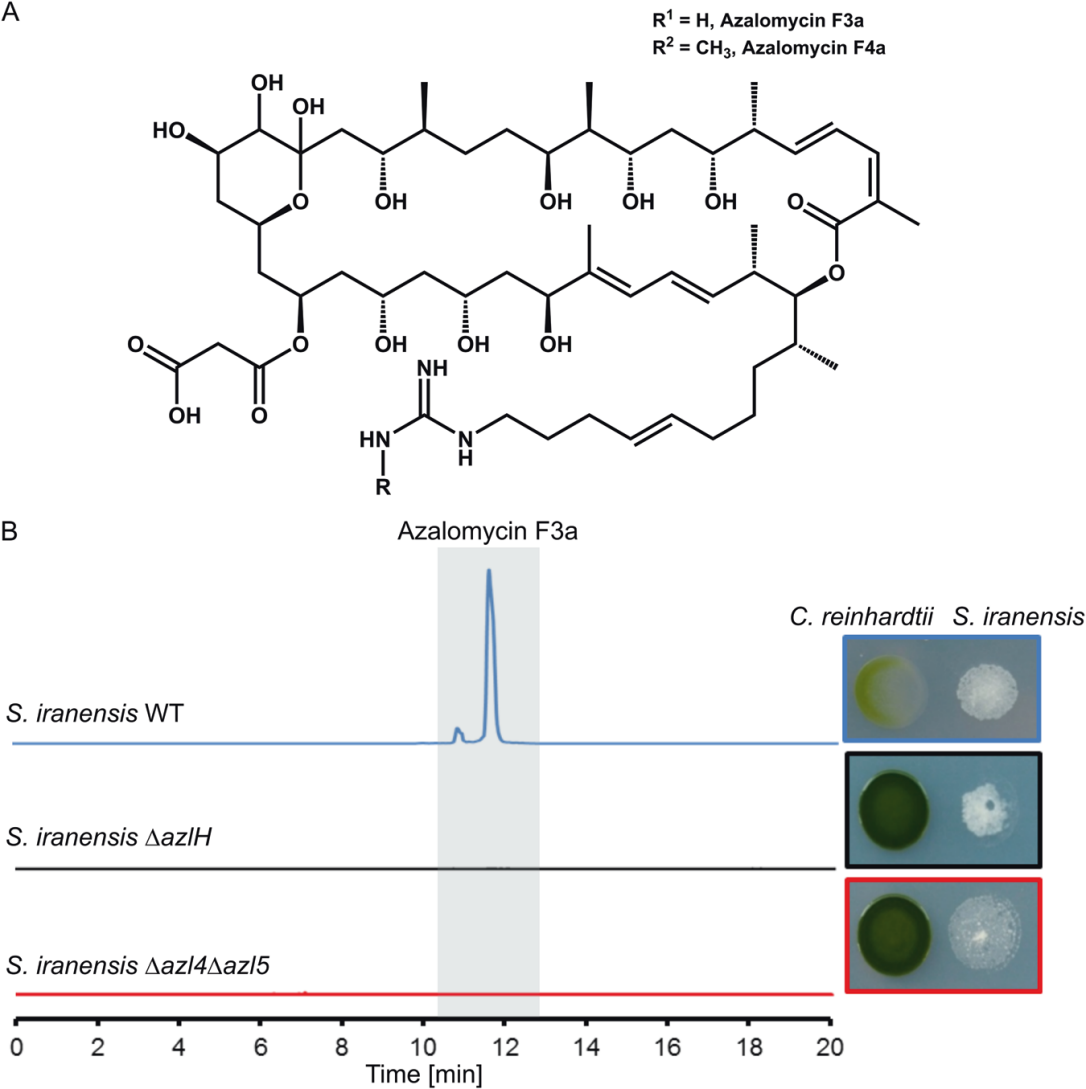
Structure of azalomycin F and its production by *S. iranensis*. **a** Azalomycin F complex consists of azalomycins F3a and F4a. **b** MS analysis in positive mode of *S. iranensis* wild type (WT)*, S. iranensis* mutants ∆*azl4*∆*azl5* and ∆*azlH*. The mutants failed to produce azalomycin F and to decolorize *C. reinhardtii* in co-culture (pictures on the right side). Extracted Ion Chromatogram: *m/z* = 1068.65–1068.80.

Since *C. reinhardtii* was decolorized over a certain distance on solid agar (Fig. 1b, c), we detected and visualized compounds that are located in the killing zone by MALDI-IMS. Two ions with *m/z* = 1069.12 and *m/z* = 1083.16 were detected (Fig. 1c). AntiSMASH analysis of the *S. iranensis* genome suggested that these two compounds could be azalomycin F3a and F4a [21, 28]. The azalomycin F complex is formed by a group of macrolactones, which were first isolated from *Streptomyces hygroscopicus* var. *azalomyceticus*. Azalomycin F consists of a 34-membered macrolactone ring and a guanidine-containing side chain (Fig. 2a, [29]) and is active against fungi as well as Gram-positive bacteria [30, 31]. A comparison of the polyketide synthase (PKS) genes (Supplementary Fig. 2) and the genes coding for tailoring enzymes (Supplementary Table 3) of the published azalomycin F biosynthetic gene cluster with the gene cluster found in *S. iranensis* supports the assumption that the two found ions indeed correspond to the azalomycin F complex. To substantiate this finding, azalomycin F-deficient mutants of *S. iranensis* were generated (Fig. 2, Supplementary Fig. 3). In the ∆*azl4*∆*azl5* mutant strain, the azalomycin F biosynthesis genes *azl4* and *azl5* were deleted. Azl4 catalyzes the activation of 4-guanidinobutyric acid by addition of coenzyme A to the molecule. Azl5 consecutively loads 4-guanidinobutyryl-CoA onto the azalomycin F PKS [32]. In the second mutant strain ∆*azlH*, the *azlH* gene, which encodes a part of the PKS [30, 32], was deleted. Neither of the mutants ∆*azl4*∆*azl5* and ∆*azlH* produced detectable amounts of azalomycin F and both failed to decolorize *C. reinhardtii* (Fig. 2b). MALDI-IMS showed that azalomycin F was exclusively present inside the bacterial colony in monoculture of *S. iranensis*, while it was released in co-culture of the bacterium with *C. reinhardtii* (Fig. 1c). We conclude that azalomycin F is the sole algicidal agent that is present under our culturing conditions and that *S. iranensis* produces and releases it in the presence of *C. reinhardtii*.

### Azalomycin F-induced algal decolorization is light dependent

We observed that *C. reinhardtii* was not decolorized when it was co-cultivated with *S. iranensis* in the dark (Fig. 1a, b). This was not due to lack of release of azalomycin F because MALDI-IMS showed that azalomycin F was still released by *S. iranensis* in the presence of *C. reinhardtii* in the dark (Supplementary Fig. 4). To provide evidence for a light-dependent algicidal activity of azalomycin F, *C. reinhardtii* cells were stained with SYTOX Blue after treatment with the algicide under light and in the dark. SYTOX Blue is a non-permeating dye that exhibits fluorescence upon binding to nucleic acids. Therefore, blue fluorescence is only detectable when cell membranes are leaky in damaged or dead cells. A positive SYTOX Blue signal was only detected when *C. reinhardtii* was treated with azalomycin F under illumination (Supplementary Fig. 5). Azalomycin F-treated cells cultured in the dark maintained their red autofluorescence, indicating that *C. reinhardtii* was not killed by azalomycin F in the dark. To analyze whether the effect of azalomycin F in light is connected to photosynthesis, we investigated the *C. reinhardtii* mutants cc4147 FuD7 (*psbA* deletion) and cc4385 (*psbD* deletion) (Supplementary Fig. 6). *psbA* and *psbD* code for the D1 and D2 reaction center proteins of photosystem II, respectively [33, 34]. As shown in Supplementary Fig. 6, azalomycin F also had a lethal effect on both mutant strains indicating that photosynthesis is not required for its lethal activity. To investigate the spectrum of algae affected by azalomycin F, we expanded our studies to microalgae belonging to different classes. Both *Euglena gracilis* and *Haematococcus pluvialis* were susceptible to azalomycin F as indicated by decolorization, a positive SYTOX blue signal and rounding up of the cells (Supplementary Fig. 7). Thus, a wide range of microalgae are susceptible to azalomycin F.

### Association of *Aspergillus nidulans* and *Chlamydomonas reinhardtii* increased algal survival in presence of azalomycin F

**Fig. 3:**
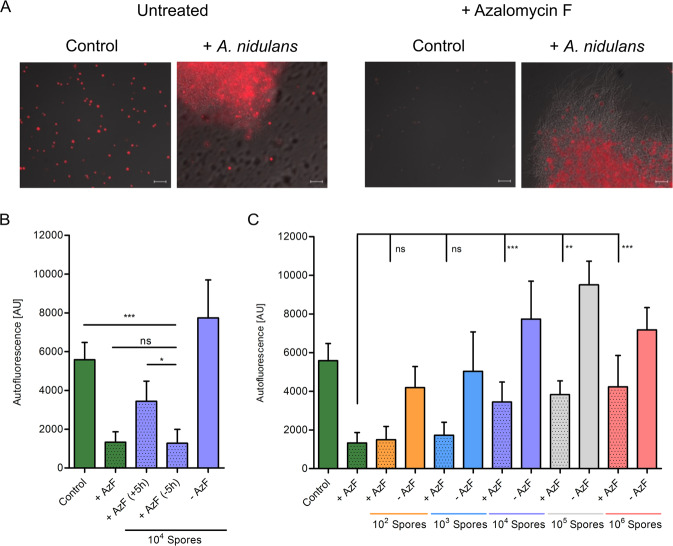
Co-cultivation of *C. reinhardtii* and *A. nidulans* protects the alga from azalomycin F. **a** Microscopy pictures of *C. reinhardtii* co-cultivated with or without *A. nidulans* and treated with or without azalomycin F. Size bars: 50 µm. **b** Autofluorescence of *C. reinhardtii* treated with azalomycin F (crosshatched bars) in monoculture (Control) and co-culture with *A. nidulans*. Loss of red autofluorescence indicates cell death. Scale bar 50 µm. **b**, **c** Autofluorescence of *C. reinhardtii*. **b** *C. reinhardtii* was either immediately treated with azalomycin F (−5 h) or allowed to associate with *A. nidulans* 5 h prior to azalomycin F addition (+5 h). **c** Correlation of protection of *C. reinhardtii* with an increased number of spores inoculated into the medium 18 h prior to addition of *C. reinhardtii*. Azalomycin F was added 5 h after coincubation. *P ≤ 0.05; **P ≤ 0.01; ***P ≤ 0.001; ns not significant; data obtained from at least three biological replicates, error bars represent SDs.

We observed *C. reinhardtii* cells that still appeared green and alive in a tripartite interaction with *A. nidulans* and *S. iranensis* (Supplementary Fig. 8). These data imply that presence of *A. nidulans* reduces the algicidal activity of *S. iranensis*. We therefore analyzed whether *A. nidulans* protects *C. reinhardtii* against purified azalomycin F, since the fungus proved to be more resistant to azalomycin F than the alga (Supplementary Fig. 9). For this purpose, we co-cultivated *A. nidulans* with *C. reinhardtii* and allowed the alga to swim into the mycelia before azalomycin F was added to the co-culture. Viability of *C. reinhardtii* was measured by chlorophyll autofluorescence (Fig. 3a). As shown in Fig. 3b, *C. reinhardtii* cells that were allowed to migrate into fungal mycelia were less affected by azalomycin F compared with *C. reinhardtii* cells that were not given the time to do so. Increasing initial fungal spore inocula were applied prior to addition of *C. reinhardtii*, azalomycin F treatment and autofluorescence measurement (Fig. 3c). A higher number of spores should lead to a higher fungal biomass and increased fungal biomass correlated with better protection, as demonstrated by titrating out the effect of azalomycin F in the presence of 10^4^ spores and above (Fig. 3c). In order to estimate the specificity of this protection, we tested further fungal species. Supplementary Fig. 10 shows that *S. cerevisiae* attracts and protects *C. reinhardtii* from azalomycin F as well. A tight association, as seen with *A. nidulans*, was not observed (Supplementary Fig. 11). *Sordaria macrospora*, an important fungus to study sexual development [35], however, failed to protect *C. reinhardtii*, although the fungus attracted the alga and formed associations comparable to *A. nidulans* (Supplementary Figs. 10, 11). Our data thus suggest that the protection is associated with self-resistance, since only fungi that are resistant against azalomycin F (Supplementary Fig. 9) protect *C. reinhardtii*.

### Protection of *C. reinhardtii* by *A. nidulans* against azalomycin F can be neutralized by fungal polar lipids

**Fig. 4:**
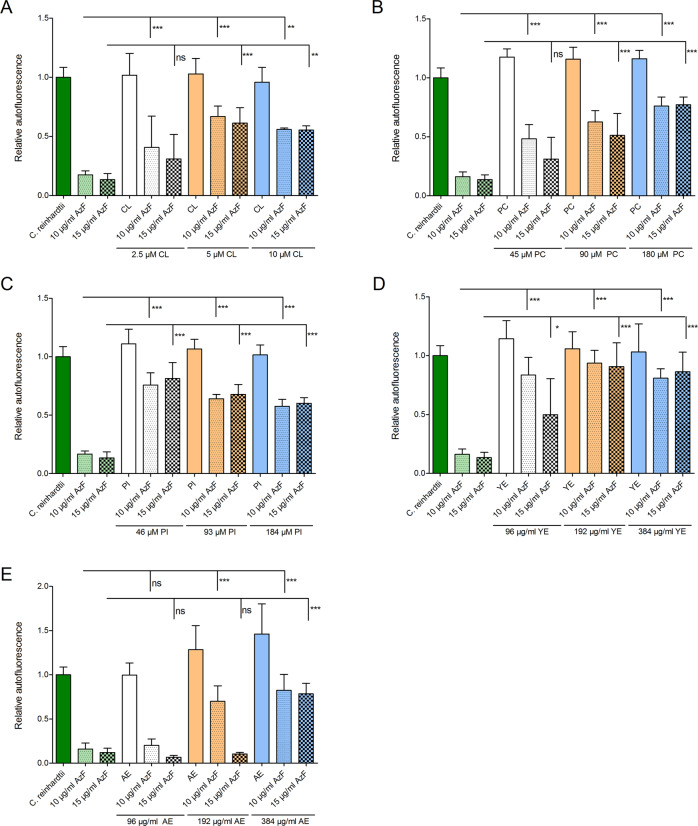
Influence of lipids on the algicidal activity of azalomycin F measured by *C. reinhardtii* autofluorescence. Algal cells were treated with 0, 10, and 15 µg/mL azalomycin F and various concentrations of polar lipids. **a** CL, cardiolipin; **b** PC, phosphatidylcholin; **c** PI, phosphatidylinositol; **d** YE, extract of polar lipids of *S. cerevisiae*; **e** AE, extract of polar lipids of *A. nidulans*. *P ≤ 0.05; **P ≤ 0.01; ***P ≤ 0.001; ns not significant; data were obtained from at least three biological replicates, error bars represent SDs.

To elucidate the mechanisms underlying the protection of *C. reinhardtii*, against azalomycin F by *A. nidulans*, we sought to identify the target of azalomycin F. Recently, the binding of azalomycin F5a to membrane components of methicillin-resistant *Staphylococcus aureus* was proposed as a determinant of its antibacterial activity [26, 36]. Consequently, we reasoned that the addition of lipids to the culture medium could neutralize the algicidal activity of azalomycin F. As shown in Fig. 4a–d, cardiolipin (CL), 16:0-i15:0 phosphatidylcholin (PC), phosphatidylinositol (PI), and a commercially available extract of polar lipids of *S. cerevisiae* (YE) all significantly lowered the detrimental effect of azalomycin F on *C. reinhardtii*. Since 1,2-dipalmitoyl-*sn*-glycero-3-phosphoglycerol (DPPG) and the sphingolipid d18:1/18:0 C18 glucosyl(β)ceramide (GC) did not affect the activity of azalomycin F (Supplementary Fig. 12), these findings indicate that there is binding specificity toward distinct polar lipids.

Based on these findings, we hypothesized that a polar lipid extract of *A. nidulans* should also neutralize the effect of azalomycin F on *C. reinhardtii*. Indeed, addition of *A. nidulans* polar lipids to the culture of *C. reinhardtii* prevented the algae from decolorization in a dose-dependent manner (Fig. 4e). Addition of 384 µg/mL of the polar lipid extract of *A. nidulans* (AE) to *C. reinhardtii* treated with azalomycin F restored the autofluorescence to the level of untreated controls. Thus, binding of azalomycin F to membrane polar lipids is required for its antibacterial activity [26, 36] as well as for its antifungal [37] and algicidal activity, as shown here.

### *Aspergillus nidulans* attracts and fosters the growth of *Chlamydomonas reinhardtii*

**Fig. 5:**
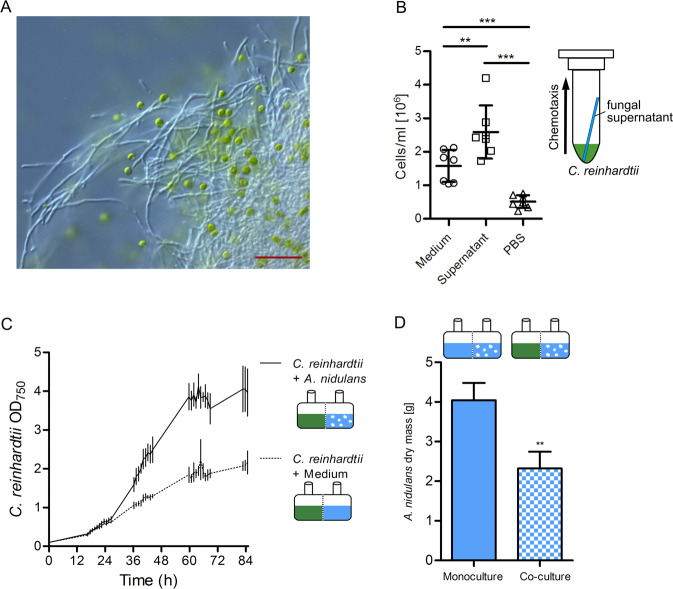
Association of *A. nidulans* with *C. reinhardtii*. **a** Microscopy picture showing *C. reinhardtii* cells (green) in mycelium of *A. nidulans*. Scale bar: 50 µm. **b** Numbers of *C. reinhardtii* cells found in capillaries filled with culture supernatant of *A. nidulans*, medium or PBS. ** represents *P* ≤ 0.01, ***P ≤ 0.001, calculated from at least three biological replicates; error bars indicate SDs. **c** Optical density of *C. reinhardtii* cells reached after co-cultivation with *A. nidulans* in co-cultivation chambers, separated by a PVDF membrane pore size 0.1 µm. **d** Dry masses of *A. nidulans* grown in mono- and co-culture.

We were able to show that an algal–fungal association protects *C. reinhardtii* from azalomycin F. Figure 5a shows such an association of *A. nidulans* and *C. reinhardtii*. To address the question whether this assembly is actively or passively established, a chemotaxis assay was performed. Figure 5b shows that significantly (*P* ≤ 0.01) more *C. reinhardtii* cells swam into a glass capillary loaded with supernatant of an *A. nidulans* culture than into a capillary filled with fresh medium (Fig. 5b). PBS served as a negative control showing unspecific swimming without an obvious chemotactic cue. Also supernatants of the other fungi tested, i.e., *S. cerevisiae* and *S. macrospora*, attracted the *C. reinhardtii* cells, indicating that *C. reinhardtii* actively swims toward the fungi.

In order to measure any effects on algal or fungal growth, co-cultivation chambers [25] were applied to separate the organisms by a PVDF membrane with a pore size of 0.1 µm. When co-cultured with *A. nidulans, C. reinhardtii* exhibited a more than two-fold increased growth rate (µmax = 2.5) and higher overall cell density compared with the monoculture (Fig. 5c). In contrast, *A. nidulans* produced a lower biomass in co-culture, compared with the monoculture at the end of the experiment (Fig. 5d). In the light of our data showing protection of the alga by fungal polar lipids, it was conceivable that more lipids were formed as an adaptive response and at the same time, energy would be redirected to lipid metabolism of the fungus explaining lower growth of *A. nidulans* in co-culture with *C. reinhardtii*. Therefore, we measured the expression of polar lipid biosynthesis genes of *A. nidulans* in presence of *C. reinhardtii* by qRT-PCR. As shown in Supplementary Fig. 13, overexpression of polar lipid biosynthesis genes was not observed in presence of *C. reinhardtii* excluding both possibilities. Taken together, our findings suggest a chemotactically driven attraction of the alga to the fungus, which ultimately provides benefit to the alga by protecting against toxins.

## Discussion

**Fig. 6:**
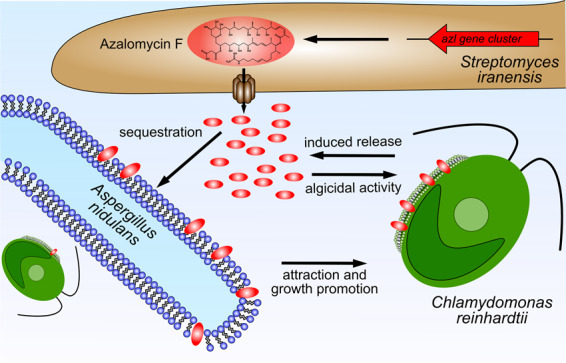
Graphical model of the tripartite interaction of *S. iranensis*, *A. nidulans*, and *C. reinhardtii*. *S. iranensis* produces algicidal azalomycin F and releases it in presence of *C. reinhardtii*. As a counter measure, *C. reinhardtii* is attracted by *A. nidulans* and takes shelter in its mycelium. *A. nidulans* provides a high surface to azalomycin F and sequesters the compound. This way less azalomycin F is accessible for *C. reinhardtii* and the alga is less affected by the compound.

Although algal–fungal co-cultures have been successfully used for biotechnological processes [38], as well as establishing algal–fungal [39] and tripartite algal–fungal–bacterial interaction systems [40], the responses of microbial consortia to harmful invaders remained largely understudied. An important step toward the goal of understanding important ecological interactions and their evolutionary selection is the study of increasingly complex biosystems in the laboratory. Among the simplest naturally occurring examples of such interactions are lichens that represent an early evolutionary emergence of organismal co-existence [3, 41]. Lichens typically consist of a photobiont, such as a green alga or a cyanobacterium and a fungal partner, the mycobiont [42]. Recent studies also reported the association of bacterial consortia with lichens [9, 10]. How such algal–fungal associations may cooperate to facilitate responses to, for instance, invasion by toxin-producing microbes is largely unknown. Here, we discovered a lichen-like association of a fungus and a green alga that helps to protect the alga against toxic bacterial compounds (Fig. 6).

### Bacterial azalomycin F exhibits anti-Gram-positive, antifungal, and algicidal activity

We have demonstrated that *S. iranensis* kills the green alga *C. reinhardtii* when both organisms were grown in co-culture. The algicidal compound was identified as the polyketide azalomycin F that also has antifungal and antibacterial activity against Gram positives [29, 31]. The reported algicidal activity found here points to a potential role of toxins in shaping algal–fungal associations.

For only a few natural products their ecological meaning has been elucidated. For example, terpenoids serve various functions ranging from chemical communication to defense against predators in plants [43]. The anaerobic bacterium *Clostridium puniceum* uses the polyphenolic metabolite clostrubin to be able to grow in an oxygen-containing environment and to defend its habitat against competitors [44]. Bacterial cyclic lipopeptides are known to play many biological roles, from promoting motility in the case of surfactin [45], to immobilize algal cells for predation [46]. A recent example by us showed that *S. rapamycinicus* induced the production of the novel compound fumigermin in *A. fumigatus*. The compound inhibits the germination of the inducing *S. rapamycinicus* [47]. Here, we identified an ecological function of a tripartite interaction between an alga and a fungus that protects the alga against a toxic bacterial natural product.

Streptomycetes are known to synthesize algicidal compounds such as NIG355, a nigericin derivative that inhibits the growth of a dinoflagellate [48] and the harmful algal bloom-forming haptophyte *Phaeocystis globosa* [49]. The observed light-dependency of the algicidal activity of azalomycin F remains obscure. The toxicity is independent of photosynthesis as photosynthesis-defective mutants of *C. reinhardtii* were effectively killed by azalomycin F. In addition, the significance of this observation for an ecological setting is unclear, as *C. reinhardtii* will almost always be illuminated to some extent in its natural habitat. It should be noted, however, that *C. reinhardtii* has at least 18 photoreceptors that absorb light in the UV as well as blue and red visible spectrum [50], and the biophysical properties and biological functions of only a few of these are known. It is conceivable that by disturbing the plasma membrane azalomycin F affects the positioning or presence of photoreceptors with a potentially lethal outcome (Fig. 6). In addition, we tested mutants that are deficient in assembly of the photosynthetic complex [33, 34]. Although the mutants still produce chlorophyll they are deficient in photosynthesis. It is thus conceivable that the presence of chlorophyll is already sufficient to render the cells susceptible to azalomycin F in a light-dependent manner.

### Azalomycin F is specifically released in the presence of *C. reinhardtii*

Azalomycin F is produced by *S. iranensis* in monoculture but the majority of the compound is associated with the bacterial cells and not in the supernatant. Only co-cultivation of *S. iranensis* with *C. reinhardtii* triggered the release of azalomycin F, which, in turn, led to the killing of the alga. It is yet unclear whether azalomycin F resides inside the cell and is exported, or, whether it is attached to the bacterial membrane and simply detached from it upon a certain signal.

Typically, biosynthesis of an antibiotic is directly coupled to its export to avoid self-intoxication. For example, the exporters of the gyrase inhibitors simocyclinone [51] or of actinorhodin [52] are co-expressed with the corresponding biosynthesis genes. The export machinery of azalomycin F is as yet unknown as no putative exporter is encoded in the biosynthetic gene cluster [21]. That a mechanism of detection of the alga and subsequent active release, secretion or detachement of azalomycin F must be at work derives from our observation that *C. reinhardtii* is killed in conditions that prevent cellular contact.

### Azalomycin F activity is neutralized by the presence of fungal membranes

We observed that *C. reinhardtii* is protected against bacterial azalomycin F by taking shelter in the mycelium of *A. nidulans*. Furthermore, we provide evidence that lipid binding is a means of neutralizing azalomycin F algicidal activity [26, 36]. We showed that azalomycin F has specificity for some polar lipids and that the polar membrane lipid phosphatidylinositol, which is important for signaling in eukaryotic cells [53–55], is also bound by azalomycin F. Ergosterol, an important cell membrane component of both *A. nidulans* and *C. reinhardtii* [56, 57], is not bound by azalomycin F, which further underlines its specificity to polar lipids (Supplementary Fig. 12). This differs markedly from other antifungal macrolides such as amphotericin B, which is considered to primarily bind ergosterol [58]. We thus propose that polar membrane lipids are the target of azalomycin F as well as the key to the protective mechanism by competitive binding (Fig. 6). A similar strategy has been applied to neutralize bacterial toxins as an antibiotic adjunctive therapy in severe pneumococcal pneumonia. Liposomal nanoparticles acted as traps for a broad panel of bacterial toxins that are known to be inserted in cellular membranes [59, 60]. Thus, this medical concept, which is promising to treat infectious diseases, appears to have existed in nature for a long time.

It is interesting to note that *S. macrospora* could not protect *C. reinhardtii* from azalomycin F despite attracting the alga. This coincides with susceptibility of *S. macrospora* against azalomycin F that is not observed for *S. cerevisiae* and *A. nidulans*. It is tempting to speculate that only fungi resistant against azalomycin F have the capability to protect *C. reinhardtii*. The reason for the susceptibility of *S. macrospora* against azalomycin F awaits further clarification.

Fungal hyphae can provide various benefits to partner organisms, as exemplified by the symbiosis of fungi and terrestrial plants in the Mycorrhiza. Here, the fungus gains fixed carbon products from the phytobiont, in exchange provides nitrogen, phosphate, and increased water supply to the land plant [61]. The fungal hyphae can be used as “fungal highways” by predatory bacteria to bridge unfavorable environments and find new, otherwise inaccessible prey [62]. Fungal mycelia have been also suggested to form microhabitats for bacteria. For example, *Morchella crassipes* fosters growth of associated *Pseudomonas putida* bacteria that in turn were also used as a carbon source by the fungus [63]. Another example is the bacterial community colonizing the truffle-fruiting body, which produces thiophene volatiles. These compounds might form the characteristic aroma of the truffle and are discussed to attract endozoochoric spore dispersers [64]. Similarly, we found that *A. nidulans* enhanced the growth of *C. reinhardtii*.

### Algal–fungal association as a novel way of coping with biotic stress

Lutzoni et al. proposed that the common ancestor of the modern Eurotiales, including the genus *Aspergillus*, once was lichen forming but the recent fungi have lost this ability during the course of evolution [65]. However, our data indicate that there is residual capability of forming symbiosis in the filamentous fungi investigated here because, the motile single-celled green alga *C. reinhardtii* readily migrated into *A. nidulans* and *S. macrospora* hyphal shells in liquid and formed macroscopic structures, in which the algal cells gathered around fungal hyphae. Likewise, it was reported that auxotrophic mutants of *A. nidulans* unable to assimilate nitrite formed an obligate mutualism with *C. reinhardtii*. The algal partner was able to provide nitrogen to the fungus [19]. *A. nidulans* and *C. reinhardtii* may well have retained the capabilities of their lichen-forming ancestors to form a kind of algal–fungal mutualism. For *C. reinhardtii*, this is not surprising because species of the genus *Chlamydomonas* have been previously identified as mycetobiont algae in lichen-like symbiotic associations of wood-decaying fungi [66].

With reference to the co-evolution of fungi, algae, and terrestrial plants, Delaux et al. [67] postulated that plant genes relevant for symbiosis were already present in an algal ancestor. It is thus conceivable that a fungal ancestor of mycorrhiza and an ancient lichen-forming *Aspergillus* species likewise might have already possessed the genes promoting symbiosis. Indeed, it has been suggested that the fungal spread on land and the radiation of terrestrial plants are linked to their preexisting ability to establish symbioses [68]. *A. nidulans* and *C. reinhardtii* may thus have retained some genetic traits allowing for algal–fungal mutualism that provides a selective advantage over noncooperators, although both microorganisms no longer contribute to the formation of stable lichens and live solitarily.

### Protection against environmental toxins may contribute to the evolution of lichen-like associations

*C. reinhardtii* cells located within the *A. nidulans* mycelium exhibited increased survival rates when treated with azalomycin F compared with *C. reinhardtii* that was prevented from entering the fungal mycelium. Presently, abiotic factors, such as water availability and radiation, are primarily considered as triggers for tight interactions [69, 70]. It stands to reason that at some point during algal–fungal co-evolution, attacks by invasive and harmful microorganisms will have occurred. Indeed, metagenomic data have indicated the presence of virulence-associated functions within the lichen microbial consortia, such as antibiotic biosynthesis genes and secretion systems. Furthermore, streptomycetes that produce antibiotic [11, 13, 14] and antifungal metabolites [12] were successfully isolated from lichens. Our data suggest that the ability to form algal–fungal associations is a viable strategy for coping with environmental toxins and biotic challenges by providing a selection advantage based on establishing symbiosis.

## Supplementary information

Supplemental Information
