# Supplementary material for: Lichen-like association of Chlamydomonas reinhardtii and Aspergillus nidulans protects algal cells from bacteria
Source: ISME J. 2020 Aug 4;14(11):2794–805. doi: 10.1038/s41396-020-0731-2 (PMC7784976; doi:10.1038/s41396-020-0731-2)
Supplement: Supplementary file 1 — Supplemental Information [file 41396_2020_731_MOESM1_ESM.docx]

**Supplementary information**

1. **Supplementary Methods**
   1. Culture methods and strain maintenance
   2. Co-cultivation of *Aspergillus nidulans* and *S. iranensis* for *ors*-gene cluster activation
   3. Azalomycin F-deficient *Streptomyces iranensis* mutants
      1. Creation of azalomycin F-deficient *S. iranensis* mutants
      2. Verification of gene deletion of *S. iranensis* mutants
      3. Azalomycin F production, extraction and measurement
   4. Cultivation, azalomycin F treatment and SYTOX blue staining of different algae as well as *C. reinhardtii* mutants deficient in photosynthesis
      1. *Euglena gracilis* SAG 1224-5/25
      2. *Haematococcus pluvialis* CCAP 34/8
      3. *C. reinhardtii* mutants deficient in photosynthesis
      4. SYTOX Blue staining
   5. Matrix-assisted laser desorption ionization mass spectrometry imaging (MALDI-IMS)
   6. Testing of influence of azalomycin F on *C. reinhardtii* and various fungi
   7. Ergosterol pre-complexation with amphotericin B and azalomycin F
   8. Extraction of polar lipids from *Aspergillus nidulans*
   9. Total RNA extraction from *Aspergillus nidulans* and qRT-PCR
2. **Supplementary Figures, Tables and Results**
   1. Natural product formation of *A. nidulans* in co-culture with *S. iranensis*
   2. Comparison of biosynthetic gene clusters
   3. Verification of azalomycin F-deficient *S. iranensis* deletion mutants
   4. MALDI-IMS of *C. reinhardtii* and *S. iranensis* in light and in dark
   5. Light dependence of azalomycin F-mediated cytoplasmic membrane permeabilization
   6. *C. reinhardtii* photosynthesis deficient mutants
   7. Cytoplasmic membrane permeabilization of algae from different phylogenetic backgrounds
   8. Tripartite co-cultivation of *S. iranensis*, *A. nidulans* and *C. reinhardtii*
   9. Influence of azalomycin F on *C. reinhardtii* and various fungi
   10. Test of capability of *Saccharomyces cerevisiae* and *Sordaria macrospora* to attract and protect *C. reinhardtii*
   11. Association of S. cerevisiae and *S. macrospora* with *C. reinhardtii*
   12. Supplementary data on non-protecting polar lipids and amphotericin B precomplexation
   13. qRT-PCR of genes involved in lipid biosynthesis of *A. nidulans*

References

- 1. Cultures and strain maintenance

Organisms and strains used in this study are listed in Supplementary Table 1. *Streptomyces iranensis* DSM41954 (HM35^T^) was routinely grown in TSB medium, at 28 °C and 180 rpm in an Erlenmeyer flask. Typically, 5⋅10^8^ spores were inoculated into 50 mL medium to obtain a densely grown culture. Spores were generated by plating 200 µL of a densely grown culture on oatmeal agar and incubation for 14 days at 28 °C.

*Chlamydomonas reinhardtii* SAG73.72 was inoculated into TAP medium (Gorman and Levine 1965) by scraping colonies from an agar plate. Cultures were incubated at 26 °C with 120 rpm and an illumination of 30 µE m^-2^ s^-1^. All cultivations in dark were carried out in the same incubator, but plates were covered with aluminium foil. TAP agar plates with *C. reinhardtii* strains were maintained at room temperature.

*Aspergillus nidulans* RMS011 was cultivated in 50 mL AMM (Brakhage and Van den Brulle 1995), supplemented with 5 mM arginine, 1 mL/L trace elements, 0.3 mM FeSO_4_ and 0.0003 % (w/v) *p*-aminobenzoic acid (PABA). After inoculation of 10^8^ spores, cultures were incubated at 37 °C with 200 rpm. Spores were obtained from colonies grown on AMM agar plates at 37 °C for 3 days, supplemented as described for AMM.

*Saccharomyces cerevisiae* DSM70499 was grown in a preculture in YM medium (3 g/L yeast extract, 3 g/L malt extract, 5 g/L peptone, 10 g/L glucose) at 26 °C and 120 rpm shaking. For co-culture, cells were taken from such a preculture and inoculated into CYB Medium (Hom and Murray 2014) + 2 % (w/v) glucose and 10 mM KNO_2_ to a final OD_600_ of 1. *C. reinhardtii* was inoculated to a final OD_750_ of 1. 200 µl of this culture were given into a 96 well plate and azalomycin F was added. The co-cultures were incubated at 26 °C, 120 rpm and 30 µE m^-2^ s^-1^ and autofluorescence was measured after 24 hours. For the chemotaxis assay, *S. cerevisiae* was cultured in CY-TAP (TAP medium supplemented with 20 % (w/v) glucose, 0.4 mg/L calcium pantothenate, 0.4 mg/L niacin, 0.002 mg/L biotin, 0.002 mg/L folic acid, 0.4 mg/L 4-amino benzoic acid, 0.4 mg pyridoxine hydrochloride, 0.4 mg thiamine hydrochloride, 0.002 mg cyanocobalamin and 1 mL/L of a trace element stock containing 29. 2 g/L EDTA, 6.6 g/L KOH, 1.2 g/L MnCl_2_∙4H_2_O, 0.3 g/L ZnCl_2_, 0.06 g/L H_3_BO_3_, 0.2 g/L CoCl_2_∙6H_2_O, 0.4 g/L CuCl_2_∙2H_2_O, 0.05 g/L Na_2_MoO_4_∙2H_2_O, 0.018 g/L KBr, 0.003 g/L KI, 0.0018 g/L Na_3_VO_4_, 0.0018 g/L Na_2_SeO_3_, 4 g/L myo-inositol) at 26 °C and 120 rpm for two days before supernatant was sterile filtered.

*Sordaria macrospora* 1911-2 was obtained on a BMM agar plate (Esser 1982). For liquid cultures, ascospores were harvested in 0.9 % (w/v) NaCl solution and an Erlenmeyer flask containing 50 mL sordaria complete medium (0.3% (w/v) sucrose, 0.05% (w/v) NaCl, 0.05% (w/v) K_2_HPO_4_, 0.05% (w/v) MgSO_4_∙7H_2_0, 0.001% (w/v) FeSO_4_∙7H_2_0, 0.5% (w/v) tryptic soy broth, 0.1% (w/v) yeast extract, 0.1% (w/v) meat extract, 0.15% (w/v) dextrin, 0.5 % (w/v) sodium acetate, pH 7.0) was inoculated with 2 mL of spores. The culture was grown at 26 °C and 120 rpm for 24 hours. For co-cultivation, the mycelium was concentrated by miracloth and given to 200 µl *C. reinhardtii*, which was inoculated into TAP medium supplemented with 10 g/L glucose, 0.5 g/L MgSO_4_∙7H_2_O, 5 mM arginine, 1 mL/L trace elements, 0.3 mM FeSO_4_ and 0.0003 % (w/v) PABA. The microorganisms were co-cultivated for five hours at 26 °C, 120 rpm and 30 µE m^-2^ s^-1^ before azalomycin F was added. Then the cultures were incubated at 26 °C, 120 rpm and 30 µE m^-2^ s^-1^ again. For the chemotaxis assay, two pieces of agar of the obtained BMM culture were given into 50 mL TAP supplemented with 10 g/L glucose, 0.5 g/L MgSO_4_∙7H_2_O, 5 mM arginine, 1 mL/L trace elements, 0.3 mM FeSO_4_ and 0.0003 % (w/v) PABA and incubated at 26 °C, 120 rpm and an illumination of 30 µE m^-2^ s^-1^ for six days. The sterile supernatant of this culture was used for the chemotaxis assay. Co-cultivation of *S. macrospora* with *C. reinhardtii* was performed in 50 mL TAP medium supplemented as stated above.

Co-cultivations of *S. iranensis* and *C. reinhardtii* were carried out in TAP medium at 26 °C with 120 rpm and an illumination of 30 µE m^-2^ s^-1^. When *A. nidulans* was co-cultured with *C. reinhardtii* TAP was supplemented with 5 mM arginine, 1 mL/L trace elements, 0.3 mM FeSO_4_ and 0.0003 % (w/v) PABA. Co-cultivations of *C. reinhardtii* and *S. iranensis* on solid medium were conducted by loading of 15 µL of a *C. reinhardtii* preculture and 15 µL of *S. iranensis* mycelium, that was washed before in PBS, on 1.5% (w/v) TAP agar. Both microorganisms were located 1 cm apart from each other. If *C. reinhardtii* was also grown on agar, cells were centrifuged and subsequently adjusted to an OD_750_ of 2 in 35-40 ^o^C liquid 1% (w/v) TAP agar.

For tripartite co-cultures, TAP supplemented with 10 g/L glucose, 0.5 g/L MgSO_4_∙7H_2_O, 5 mM arginine, 1 mL/L trace elements, 0.3 mM FeSO_4_ and 0.0003 % (w/v) PABA was used to which *C. reinhardtii* was added to a final OD_750_ of 2. 2.5 mL of an *S. iranensis* preculture were added and *A. nidulans* mycelium was first filtered by miracloth and then directly added to this culture. The incubation was carried out at 26 °C, 120 rpm at 30 µE m^-2^ s^-1^.

- 1. Co-cultivation of *Aspergillus nidulans* and *S. iranensis* for *ors*-gene cluster activation

*Streptomyces iranensis* DSM41954 (HM35^T^) was cultivated as described in the cultivation section. For co-cultures *A. nidulans* mycelium filtered *via* miraocloth was added to fresh AMM supplemented with 5 mM arginine, 1 mL/L trace elements, 0.3 mM FeSO_4_ and 0.0003 % (w/v) PABA. 2.5 mL of *S. iranensis* preculture were added and it was incubated with 200 rpm at 37 °C for 16 hours. Natural products were extracted as described in Schroeckh *et al.* (2009).

- 1. Azalomycin F-deficient *Streptomyces iranensis* mutants
     1. Creation of azalomycin F-deficient *S. iranensis* mutants

Deletion of *S. iranensis* genes was carried out using the lambda red system. It allowed for in frame gene deletions by conjugation of *Escherichia coli* ET12567 pUZ8002 with *S. iranensis* essentially as described by (Netzker *et al.* 2016). In brief, genes to be deleted, as well as 2 kbp flanking regions upstream and downstream were amplified by PCR, using primers, which encoded DNA sequences for restriction digestion with *Hin*dIII-HF and *Xba*I (New England Biolabs, Frankfurt Germany). For all PCR experiments the Phusion^®^ High-Fidelity PCR Master Mix (Thermo Scientific, Waltham, USA) was used. The genes and their flanking regions were cloned into pKOSi and the thus generated plasmids pKOSi_*azl4_azl5* and pKOSi_*azlH* were then used to transform *Escherichia coli* α-Select. Next, pKOSi_*azl4_azl5* and pKOSi_*azlH* were transformed into *E. coli* BW25113 pIJ790 and subsequently, the genes to be deleted were replaced by the apramycin resistance gene *aac(3)IV* and the *oriT*. Recombination was achieved by using a DNA template obtained by PCR of the *aac(3)IV* gene, *oriT* and homologous sites at the ends generated by specific primers (see Supplementary Table 2). The thereby created plasmids pKOSi_Δ*azl4/∆azl5* and pKOSi_Δ*azlH* were transformed into non-methylating *E. coli* ET12567 pUZ8002 that was employed for conjugation with *S. iranensis.* Conjugation and subsequent steps were carried out according to (Netzker *et al.* 2016). The primers used to create *S. iranensis* Δ*azl4/*Δ*azl5* and *S. iranensis* Δ*azlH* are listed in Supplementary Table 2.

Supplementary Table 1: Organisms, strains and plasmids used in this study.

| **Organism or plasmid** | **Description; relevant genotypes** | **Reference/ Supplier** |
| --- | --- | --- |
| *Streptomyces iranensis* DSM41954 (HM35^T^) | Wild-type strain | Hamedi *et al.* (2010) |
| *Escherichia coli* α-Select | *deoR*, *recA*1, *endA*1, *relA*1, hsdR17 (r^-^_k_, m^+^_k_), *supE*44, *gyrA*96, *thi*-1, Fγ^-^, φ80*dacZ*Δ*M*15, Δ(*lacZY A* – *argFV*169) | Bioline GmbH, Luckenwalde, Germany |
| *Escherichia coli* BW25113 | Δ*araBAD*, Δ*rhaBAD* | Datsenko and Wanner (2000) |
| *Escherichia coli* ET12567 | *dam, dcm, hsdS, cat, tet* | MacNeil *et al.* (1992) |
| *Streptomyces iranensis* Δ*azl4*Δ*azl5* | Deletion of Δ*azl4* coding for a 4-guanidinobutanoate CoA ligase and deletion of Δ*azl5* coding for a 4-guanidinobutyryl-CoA:ACP acyltransferase | This work |
| *Streptomyces iranensis* Δ*azlH* | Deletion of Δ*azlH*, the gene encoding the last module of the azalomycin PKS protein | This work |
| *Aspergillus nidulans* RMS011 | *pabaA1, yA2*; Δ*argB*::*trpC*Δ*B*; *veA1, trpC801* | Stringer *et al.* (1991) |
|  |  |  |
| *Chlamydomonas reinhardtii* SAG73.72 | Wild-type strain, mt+ | Algal culture collection Göttingen |
| *Chlamydomonas reinhardtii* cc4147 FuD7 (*psbA* deletion) | Deletion of *psbA* encoding the D1 protein of PSII, mt+ | *Chlamydomonas* Resource Center, University of Minnesota; from Jacqueline Girard-Bascou, Institut de Biologie Physico-Chimique, Paris |
| *Chlamydomonas reinhardtii* cc4385 psbD del (+) | Deletion of *psbD* encoding the D2 protein of PSII, mt+ | *Chlamydomonas* Resource Center, University of Minnesota; originally from Tasios Melis, University of California, Berkeley |
| *Euglena gracilis* SAG 1224-5/25 | Wild-type strain | Algal culture collection Göttingen |
| *Haematococcus pluvialis* CCAP 34/8 | Wild-type strain | Culture Collection of Algae and Protozoa, Oban, Scotland |
| *Saccharomyces cerevisiae* DSM70499 | Wild-type strain | DSMZ-German Collection of Microorganisms and Cell Cultures Braunschweig, Germany |
| *Sordaria macrospora*  (1911-2) | Wild-type strain | Professor Stefanie Pöggeler,  Institute for Microbiology and Genetics, Georg-August-University Göttingen, Germany |
| pIJ773 | *aac(3)IV, oriT, bla* | Gust *et al.* (2003) |
| pIJ790 | *gam, bet, exo, cat, araC, rep101^ts^* | Gust *et al.* (2003) |
| pUZ8002 | *tra, neo, cos* | Paget *et al.* (1999) |
| pKOSi | *kan^R^, pSG5 replicon* | Netzker *et al.* (2016) |
| pKOSi_*azl4_azl5* | *kan^R^, pSG5 replicon, azl4, azl5,* 2 kbp flanking regions | This work |
| pKOSi*_*Δ*azl4/*Δ*azl5* | *kan^R^, pSG5 replicon, aac(3)IV, oriT,* 2 kbp flanking regions | This work |
| pKOSi_*azlH* | *kan^R^, pSG5 replicon, azlH,* 2 kbp flanking regions | This work |
| pKOSi_Δ*azlH* | *kan^R^, pSG5 replicon, aac(3)IV, oriT,* 2 kbp flanking regions | This work |

- - 1. Verification of gene deletions in *S. iranensis*

Mutants were verified by PCR amplifying the wild-type gene or the flanking region of the wild-type gene along with the antibiotic resistance marker gene. PCR experiments were conducted using the MyTaq^TM^ Red Mix (Bioline GmbH, Luckenwalde, Germany). Primers used are listed in Supplementary Table 2. The results of this verification are shown in Supplementary Figure 3.

Supplementary Table 2: Primers used for generation of deletion plasmids and for verification of mutant strains.

| **Replaced gene(s)** | **Primer (sequence) used for gene amplification and restriction enzyme used for cloning.** | **Primer (sequence) used for *aac(3)IV* amplification from pIJ773 (Gust *et al.* 2003). Bold and lower case letters: annealing sequence.** | **Primers for verification of gene deletion** |
| --- | --- | --- | --- |
| *Azl4azl5* | oTN125 (GATTGTAAGCTT ATCAGGAACGCCCACTTGTT) *Hin*dIII  oTN126 (CAGTCATCTAGA TGGCGGAGTTCGTGATGTTC) *Xba*I | oTN127 (ATTACACGG GGGCGCTGCCCGGGG GCAGCGCCTCCATCA**tgtaggctggagctgcttc**)  oTN128 (CCGGCGGCTGGACCG GGCGATGACCATGGCGGGCAC ATG**attccggggatccgtcgacc**) | For PCR of wild-type gene: oTN344 (CGATGGTCGGAAATC ATTGC) oTN345 (GGCGGATACAACATGTACAC)  For PCR of deletion: oTN344 and oTN309 (GTCCAGTCGGTCATGCCTTT) |
| *azlH* | OMK_023 (GTACTCAAG CTTGTGAACATCGTC TGATCGAG) *Hin*dIII  OMK_024 (GCATCATCT AGAGTCGAGGAACTG TCGTTCAC) *Xba*I | OMK_025 (CGCCAATCCCGACTGGAA AGA GAGTGACGGAGAACGGTG**attccggggatccgtcgacc**)  OMK_026 (CCCGCGGGCCATCGATGCGGG CGG GCGCGTGCTGCGCTA**tgtaggctggagctgcttc**) | For PCR of wild-type gene:  oTN394 (GGCGATATGCCGAAGAACTC) oTN395 (CACGATGAT GGAGAAGCAGG)  For PCR of gene deletion: oTN454 (TCGAGGCCGTTCTTG GTGCTCTGTA)  oTN455 (GGTGGTATCTGCATCTGTTCGAGGG) |

- - 1. Azalomycin F production, extraction and measurement

5∙10^8^ spores of *S. iranensis* were inoculated into 50 mL seed medium (Netzker *et al.* 2016). The culture was incubated at 30 °C, with 180 rpm for 5 days in baffled flasks. Then, 5 mL of these precultures were used to inoculate 45 mL HPM medium in baffled flasks. It was incubated at 30 °C, 180 rpm for another 5 days. The cultures were extracted with ethyl acetate. The resulting extracts were dried with sodium sulfate and concentrated under reduced pressure. The dried extract was dissolved in 1 mL MeOH. HPLC-MS measurements were performed using an Exactive Orbitrap High Performance Benchtop LC-MS with an electrospray ion source and an Accela HPLC system (Thermo Fisher Scientific, Bremen, Germany). HPLC conditions: C18 column (Betasil C18 3 µm 150 x 2.1 mm) and gradient elution (MeCN/0.1 % (v/v) HCOOH (H_2_O) 5/95 for 1 min, going up to 98/2 in 15 min, then 98/2 for another 3 min; flow rate 0.2 mL min^-1^; injection volume: 3 µL).

HPM (Oatmeal production medium): 20 g/L oatmeal, 0.1 g/L FeSO_4_∙7H_2_O, 1.7 g/L K_2_HPO_4_, 28.8 g/L sucrose, 5 g/L lysine-HCl, 2.5 g/L NaNO_3_, 0.1 g/L MgSO_4_∙7H_2_O, 0.1 g/L ZnSO_4_∙7H_2_O, pH 6.

- 1. Cultivation, treatment with azalomycin F and SYTOX blue staining of different algae and *C. reinhardtii* mutants deficient in photosynthesis
     1. *Euglena gracilis* SAG 1224-5/25

To estimate the algicidal spectrum of azalomycin F, algae belonging to different families were tested for their susceptibility. *Euglena gracilis* SAG 1224-5/25 is a representative of the family [*Euglenophyceae*](https://de.wikipedia.org/w/index.php?title=Euglenophyceae&action=edit&redlink=1) (Bicudo and Menezes 2016). It was routinely grown in 3N-BBM medium + EG (Culture collection of Algae and Protozoa, Oban, Scotland), at 26 °C, 120 rpm and 30 µE m^-2^ s^-1^ for 6 days. Then, the culture was centrifuged (2000g, 2 minutes) and adjusted in 3N-BBM + EG to a final OD_750_ of 2. 20 µg/mL azalomycin F were immediately added. The culture was incubated at 26 °C, with 120 rpm and 30 µE m^-2^ s^-1^ for 16 hours prior to SYTOX blue staining.

3N-BBM+EG: 30 mL/L 25 g/L NaNO_3_, 10 mL/L 2.5 g/L CaCl_2_∙2H_2_O, 10 mL/L 7.5 g/L MgSO_4_∙7H_2_O, 10 mL/L 7.5 g/L K_2_HPO_4_∙3H_2_O, 10 mL/L 17.5 g/L KH_2_PO_4_, 10 mL/L 2.5 g/L NaCl, 1 mL/L 1.2 g/L Thiaminhydrochloride, 1 mL/L 1 g/L cyanocobalamin, 1 g/L sodium acetate trihydrate, 1 g/L beef extract. 2 g/L tryptone, 2 g/L yeast extract, 10 mL/L of 1 g/L CaCl_2_, 6 mL/L trace element solution (97 mg/L FeCl_3_∙6H_2_O, 41 mg/L MnCl_2_∙4H_2_O, 5 mg/L ZnCl_2_, 2 mg/L CoCl_2_∙6H_2_O, 4 mg/L Na_2_MoO_4_∙2H_2_O).

- - 1. *Haematococcus pluvialis* CCAP 34/8

*Haematococcus pluvialis* CCAP 34/8 is a member of the family [*Haematococcaceae*](https://de.wikipedia.org/w/index.php?title=Haematococcaceae&action=edit&redlink=1) (Guiry and Guiry 2019, Silva 1980). The alga was cultured in 3 mL KM1 medium (Kobayashi *et al.* 1991) at 25 °C with 120 rpm and 30 µE m^-2^ s^-1^ in a 12h light and 12h dark cycle for 5 days. To test its susceptibility to azalomycin F, 200 µL of this preculture were added to a 48-well plate and treated with 20 µg/mL azalomycin F. The plate was incubated at 26 °C with 120 rpm and 30 µE m^-2^ s^-1^ constant light for 6 days prior to SYTOX blue staining.

- - 1. *C. reinhardtii* mutants deficient in photosynthesis

The observation that light seems to play an important role in azalomycin F killing of *C. reinhardtii* led to the hypothesis, that photosynthesis might be the target of this compound. Therefore, we tested two photosynthesis-deficient *C. reinhardtii* strains for their susceptibility to azalomycin F. *C. reinhardtii* cc4147 FuD7 (*psbA* deletion) and cc4385 (*psbD* deletion) were grown in TAP medium at 26 °C with 120 rpm and 30 µE m^-2^s^-1^. For azalomycin F treatment, the cultures were centrifuged and adjusted to an OD_750_ of 2. 10 µg/mL azalomycin F was added and the cultures were incubated for 16 h prior to SYTOX blue staining. A positive SYTOX Blue signal indicates a permeable cytoplasma membrane.

- - 1. SYTOX Blue staining

SYTOX Blue Dead Cell Stain was purchased from Thermo Fisher Scientific (Waltham, MA, USA). 50 µL of the culture to be tested were withdrawn and 6.25 µL of 37% (v/v) paraformaldehyde was added. Subsequently, 1 µL of 500 µM SYTOX Blue dye was added and the cells were incubated for 15 minutes at room temperature in the dark and ambient shaking on a rocking shaker. Autofluorescence was excited at 653 nm and measured at an emission of 668 nm. SYTOX blue was excited at 353 nm and emission was measured at 465 nm.

- 1. Matrix-assisted laser desorption ionization mass spectrometry imaging (MALDI-IMS)

Cultures on Indium-Tin-Oxide (ITO)-coated slides were dried for 48h at 37 °C. The dried samples were then sprayed with a saturated solution (20 mg/mL) of universal MALDI matrix (1:1 mixture of 2,5-dihydroxybenzoic acid and α-cyano-4-hydroxy-cinnamic acid; Bruker Daltonics, Bremen, Germany) prepared in acetonitrile/methanol/water (70:25:5, v/v/v), using the automatic system ImagePrep device 2.0 (Bruker Daltonics, Bremen, Germany) in 60 consecutive cycles (the sample was rotated 180° after 30 cycles) of 41 s (1 s spraying, 10 s incubation time, and 30 s of active drying) similar to Hoffmann and Dorrestein (2015). The sample was analyzed in an UltrafleXtreme MALDI TOF/TOF (Bruker Daltonics, Bremen, Germany), which was operated in positive reflector mode using flexControl 3.0. The analysis was performed in the 100-3000 Da range, with 30% laser intensity (laser type 4), accumulating 1000 shots by tanking 50 random shots at every raster position. Raster width was set at 200 µm. Calibration of the acquisition method was performed externally using Peptide Calibration Standard II (Bruker Daltonics, Bremen Germany) containing Bradykinin1-7, Angiotensin II, Angiotensin I, Substance P, Bombesin, ACTH clip1-17, ACTH clip18-39, and Somatostatin 28. Spectra were processed with baseline subtraction in flexAnalysis 3.3 and aligned using several endogenous peaks (compounds present in the culture media). Processed spectra were uploaded in flexImaging 3.0 for visualization and SCILS Lab 2015b for analysis and representation. Chemical images were obtained using Total Ion Count normalization and weak denoising.

- 1. Testing of influence of azalomycin F on *C. reinhardtii* and fungi

Cotton discs were loaded with 12 µg azalomycin F and placed on agar suited for the microorganism to be tested. *C. reinhardtii* was inoculated into TAP agar at an OD_750_ = 2 and incubated at 26 °C and 30 µE m^-2^ s^-1^. 500 spores of *A. nidulans* were spot inoculated in 1 cm distance of azalomycin F on AMM agar supplemented with 5 mM arginine, 1 mL/L trace elements, 0.3 mM FeSO_4_ and 0.0003 % (w/v) PABA and incubated at 37 °C. *S. cerevisiae* was spread on YM-Agar and the filter disc was placed in the middle of the petri dish. A cut-out piece of *S. macrospora* on BMM (Esser 1982) was placed on Medium 90 (30 g/L malt extract, 3 g/L soy peptone, 15 g/L agar) in 1 cm distance of azalomycin F and it was incubated at 26 °C.

- 1. Ergosterol pre-complexation with amphotericin B and azalomycin F

Pre-complexation of ergosterol with azalomycin F and amphotericin B was carried out essentially according to Anderson *et al.* (2014). Ergosterol was purchased from Sigma-Aldrich (St. Louis, MO, USA). In short, 25 µL of a 4 mg/mL stock solution of ergosterol in chloroform were evaporated in a glass vial under a gentle stream of nitrogen. The residual ergosterol was solved in 1 mL of 50 µM amphotericin B or azalomycin F dissolved in DMSO. The vials were heated to 80 °C for 1h to dissolve the ergosterol. As controls, azalomycin F and amphotericin B were heated, but no ergosterol was added. Then, the vials were incubated at 25 °C under gentle shaking to allow complexation. The experimental as well as the control samples were added to *C. reinhardtii* at an OD_750_ of 2. It was incubated at 26 °C with 120 rpm and 30 µE m^-2^ s^-1^. Autofluorescence was measured on a Tecan M200 pro microplate reader (Tecan Trading AG, Männedorf, Switzerland) after 48 h using an excitation wavelength of 480 nm and measuring the emission at 684 nm.

- 1. Extraction of polar lipids from *Aspergillus nidulans*

Polar lipids of *A. nidulans* were extracted according to the method of Birch *et al.* (1998).

- 1. Total RNA extraction from *Aspergillus nidulans* and qRT-PCR

Total RNA was purified using the Universal RNA Purification Kit (Roboklon, Berlin, Germany). Reverse transcription of 2 µg RNA was performed with Maxima Reverse Transcriptase (Thermo Fisher Scientific, Darmstadt, Germany) for 3 hours at 48 °C.

qRT-PCR was carried out as described in Schroeckh *et al.* (2009). As an internal control, the fungal gene for gamma actin (*AN6542*) was used for comparison of expression levels as described in Schroeckh *et al.* (2009). Primers used for qRT-PCR are listed below:

| Target gene and direction | Sequence |
| --- | --- |
| AN0913_forward | TTCGCAAGTCCTTGGAGTGC |
| AN0913_reverse | GCCATAACTGGGAACGAGACTG |
| AN4332_forward | CGCAGTGACAGTGAAGAGAAGG |
| AN4332_reverse | TCCGTTTCGACGCCATTCAG |
| AN6530_forward | CCAACACTTGGTCTTCCTGACG |
| AN6530_reverse | TGTAGGCATTGTGACCACGC |
| AN6580_forward | ACGGCGATATAACCTCCAGACC |
| AN6580_reverse | TAACGGCAAGACAAGCGACG |

1. Supplementary Figures, Tables and Results
   1. Natural product formation of *A. nidulans* in co-culture with *S. iranensis*


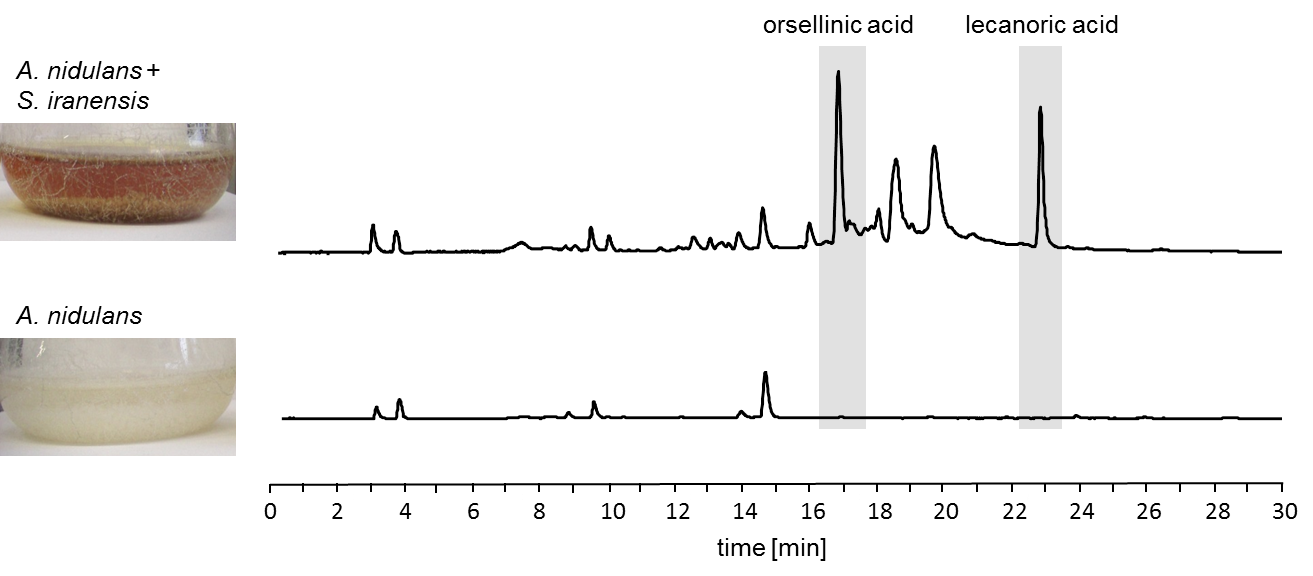


Supplementary Figure 1: Co-cultivation of *A. nidulans* and *S. iranensis* leads to production of orsellinic acid and lecanoric acid by the fungus. Co-Cultivation of *A. nidulans* and *S. iranensis* (top) in comparison to an *A. nidulans* monoculture (bottom). On the left, flasks with the cultures are shown. The co-culture shows a brown coloration, while the monoculture of *A. nidulans* remains uncolored. On the right, the MS spectra show the typical products of the *ors*-biosynthetic gene cluster, *i.e*., orsellinic acid and lecanoric acid. Therefore, also *S. iranensis* is able to induce the *ors*-gene cluster in *A. nidulans*, as well as its close relative *S. rapamycinicus* (Schroeckh *et al.* 2009).

- 1. Comparison of biosynthetic gene clusters


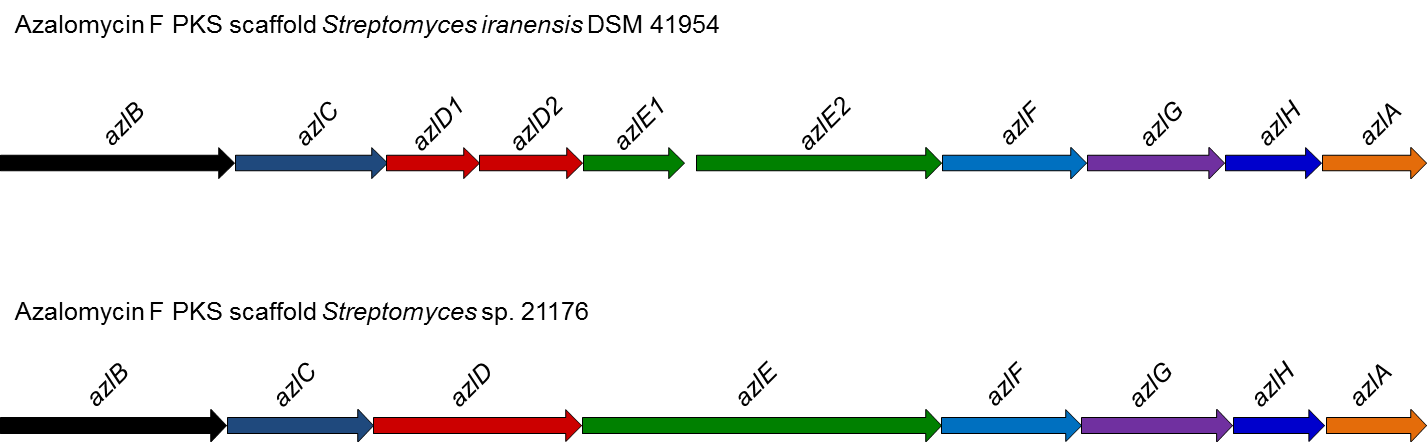


Supplementary Figure 2: Comparison of the azalomycin F PKS scaffold between *S. iranensis* DSM41954 (HM35^T^) and *Streptomyces* sp. 21176. Apparently, the organization of the genes encoding the PKSs is highly similar. However, in *Streptomyces* sp. 21176 there are two PKS genes, *i.e*., *azlD* and *azlE*, that were both split in *S. iranensis* in two genes, *i.e*., *azlD1*, *azlD2*, *azlE1*, and *azlE2* (Xu *et al.* 2017).

| **Gene** | **Putative function of gene product** | **Gene in *Streptomyces iranensis* DSM41954 (HM35^T^)** | **Similarity to protein in *Streptomyces* sp. 211726 coverage (%) /similarity (%)** |
| --- | --- | --- | --- |
| *azl1* | membrane protein | *SIRAN1046* | 55 / 93 |
| *azl2* | cytochrome P450 | *SIRAN1045* | 92 / 98 |
| *azl3* | ferredoxin | *SIRAN1044* | 100 / 97 |
| *azl4* | 4-guanidinobutanoate:CoA ligase | *SIRAN1023* | 100 / 99 |
| *azl5* | 4-guanidinobutyryl-CoA:ACP acyltransferase | *SIRAN1022* | 96 / 94 |
| *azl6* | TetR-family transcriptional regulator | *SIRAN1021* | 98 / 96 |
| *azl7* | hydrolase | *SIRAN1020* | 100 / 95 |
| *azl8* | Hxl-family transcriptional regulator | *SIRAN1019* | 100 / 99 |
| *azl9* | endoribonuclease L-PSP | *SIRAN1018* | 100 / 98 |
| *azl10* | cellulose-binding family II | *SIRAN1017* | 100 / 97 |
| *-* | - | *SIRAN1016* | - |
| *azl11* | hypothetical protein | *SIRAN1015* | 100 / 98 |
| *azl12* | GntR-family transcriptional regulator | *SIRAN1014* | 95 / 95 |
| *azl13* | 4-guanidinobutyramide hydrolase | *SIRAN1013* | 100 / 98 |
| *azl14* | amino acid permease | *SIRAN1012* | 99 / 95 |
| *azl15* | AraC-family transcriptional regulator | *SIRAN1011* | 100 / 97 |

Supplementary Table 3: *Streptomyces iranensis* DSM41954 (HM35^T^) comparison of the azalomycin F biosynthetic genes encoding tailoring enzymes to *Streptomyces* sp. 211726 .

- 1. Verification of azalomycin F-deficient *S. iranensis* deletion mutants


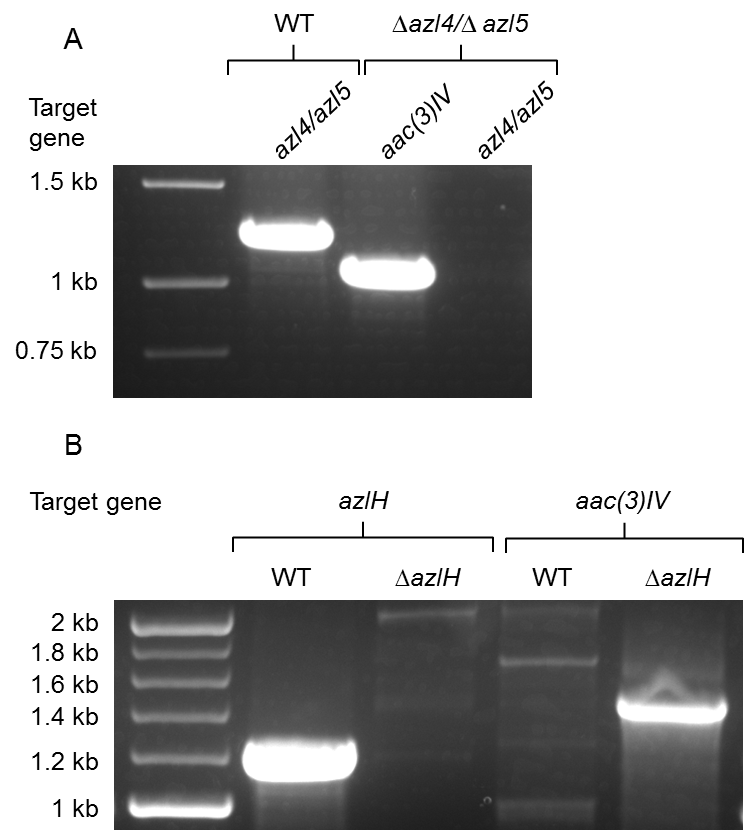


WT

∆*azl4*/∆*azl5*

Supplementary Figure 3: Deletion of *azl4*/*azl5* (A) and *azlH* (B)*.* A. Lanes *azl4/azl5* depict a PCR product obtained by primers targeting the wild-type *azl4* and *azl5* genes (1202 bp). Lane *aac(3)IV* shows a PCR product obtained from primers targeting the resistance marker (1035 bp). The target organisms are stated on top. *S. iranensis* ∆*azl4*/∆*azl5* is positive for the resistance marker (*aac(3)IV*) and negative for the wild-type genes*.* B. Lanes WT depict PCR products generated from wild-type gDNA, while lanes ∆*azlH* show PCR products of the deletion mutant. The genes named on top are the PCR target genes. The wild type generated a positive signal for the PCR on wild-type *azlH* gene (1176 bp) and no signal for the PCR targeting the resistance marker gene *aac(3)IV*. In contrast, the deletion mutant did not show a signal for wild-type *azlH*, but a clear band for *aac(3)IV* (1449 bp).

- 1. MALDI-IMS of *C. reinhardtii* and *S. iranensis* in light and in dark


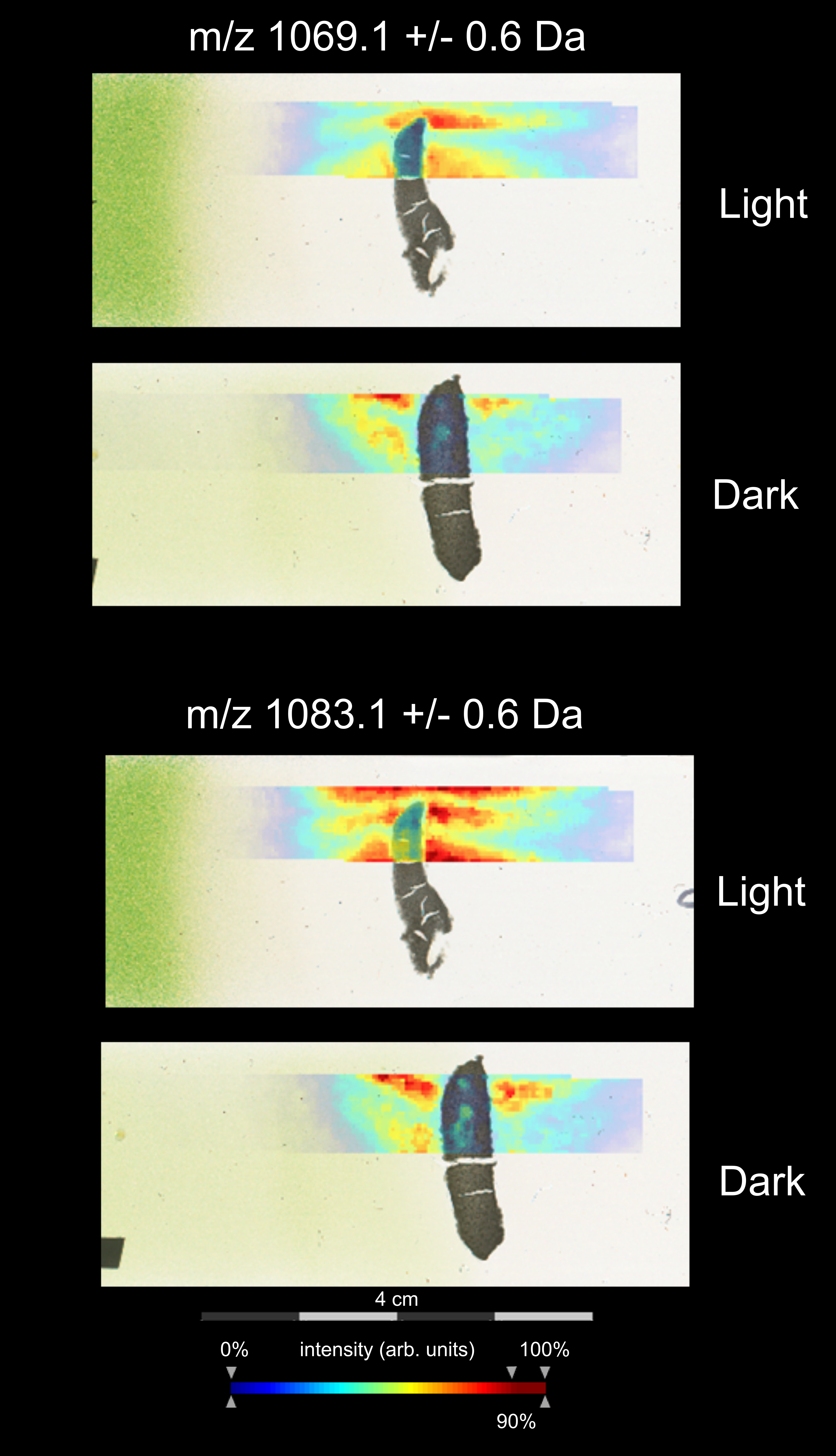


Supplemenary Figure 4: Azalomycin F is released by *S. iranensis* in dark as well as in light when in contact with *C. reinhardtii*. MALDI-IMS images indicate color-coded abundance of ion *m/z* = 1069.1 +/-0.6Da (azalomycin F3a, [M+H]^+^) and ion *m/z* = 1083.1 +/- 0.6 Da (azalomycin F4a, [M+H]^+^). Color code: blue = low abundance, red = high abundance of ion.

- 1.
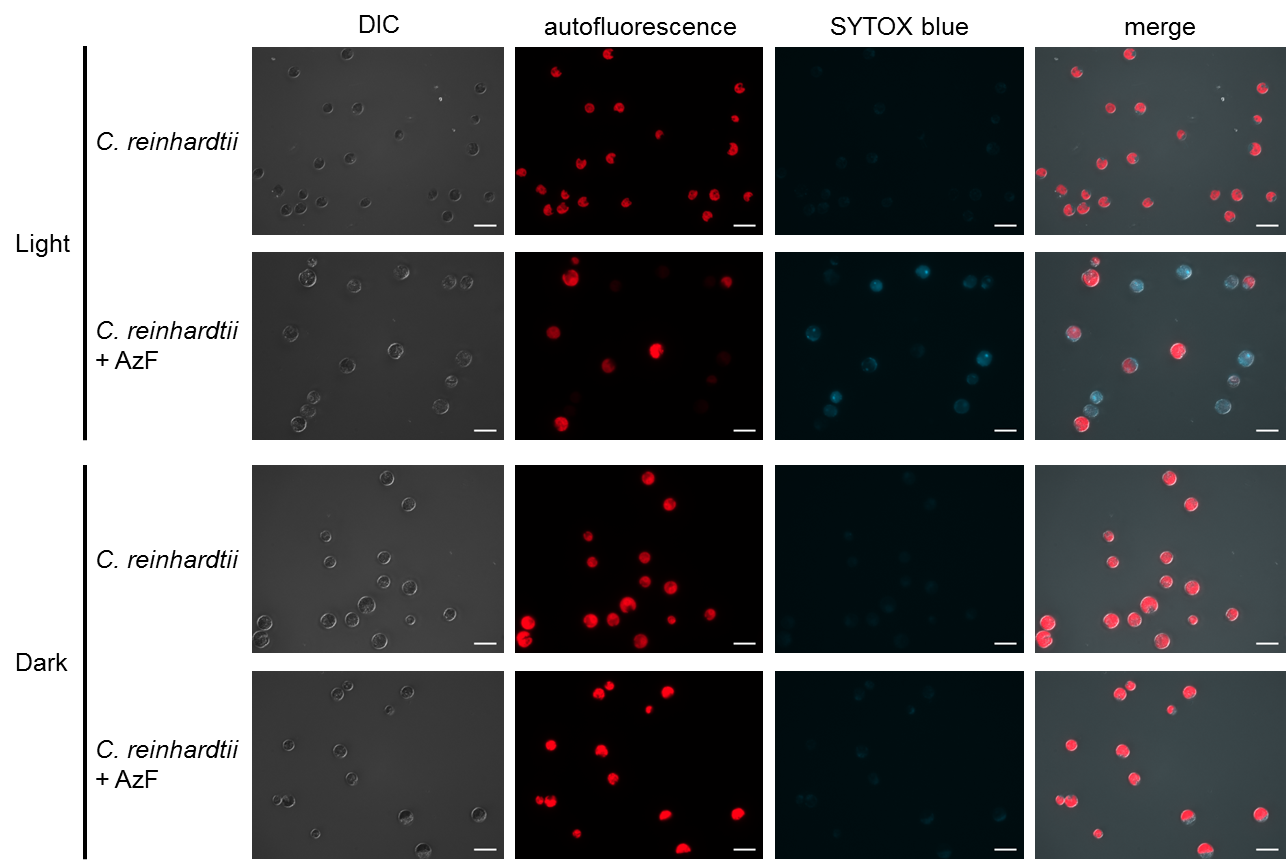
Light dependence of azalomycin F-mediated cytoplasmic membrane permeabilization

Supplementary Figure 5: Azalomycin F-mediated killing of *C. reinhardtii* in light and in dark. Red autofluorescence indicates live cells, positive SYTOX Blue signal marks penetration of the dye into the cells, which indicates increased membrane permeability and cell death. AzF: 5 µg/ml azalomycin F. Scale bars: 20 µm.

- 1.
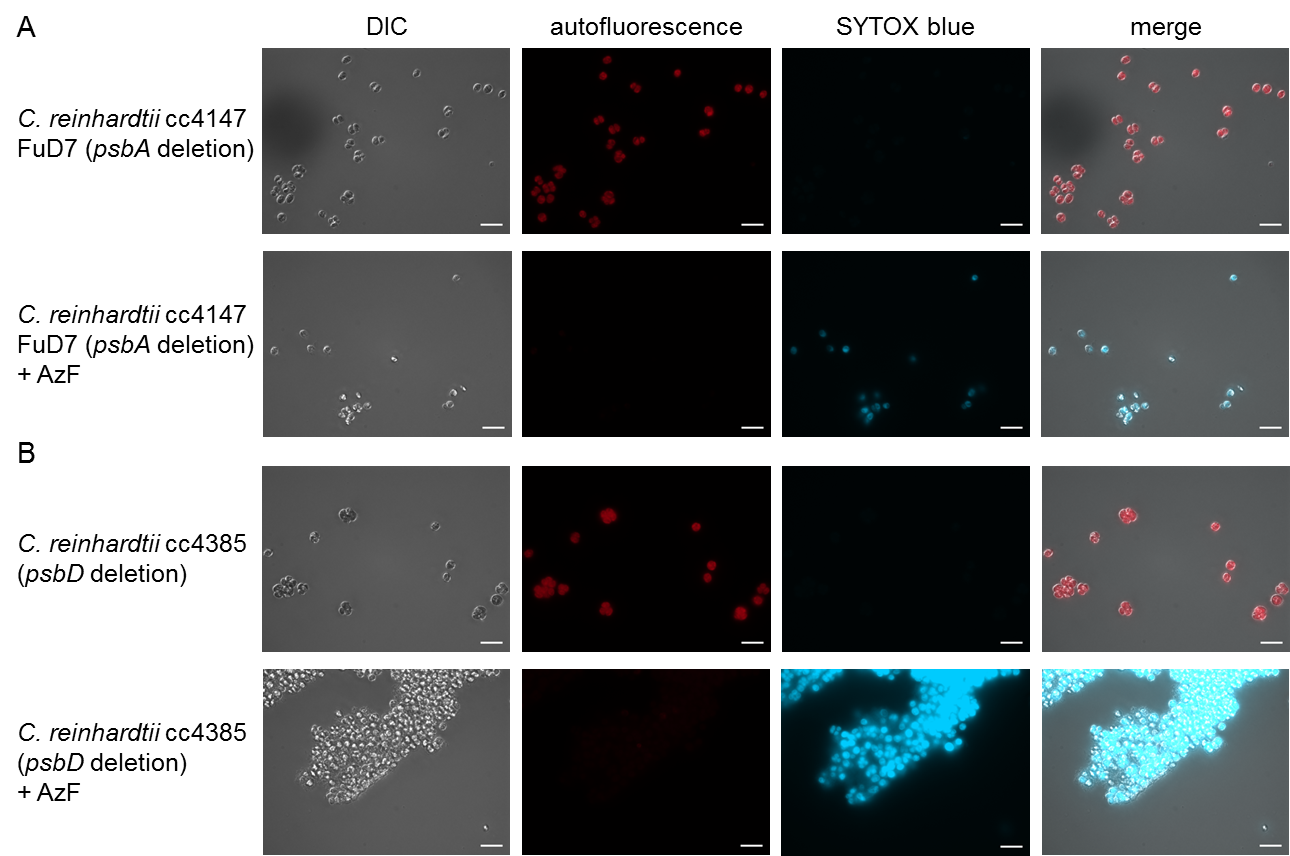
*C. reinhardtii* photosynthesis deficient mutants

Supplementary Figure 6: Cell permeabilization by azalomycin F of photosynthesis-deficient *C. reinhardtii* mutants.

A positive autofluorescence and SYTOX Blue signal indicate live cells and cell death, respectively. A. *C. reinhardtii* cc4147 FuD7 (*psbA* deletion) B. *C. reinhardtii* cc4385 (*psbD* deletion). 20 µg/ml AzF. Scale bars: 20 µm.

- 1.
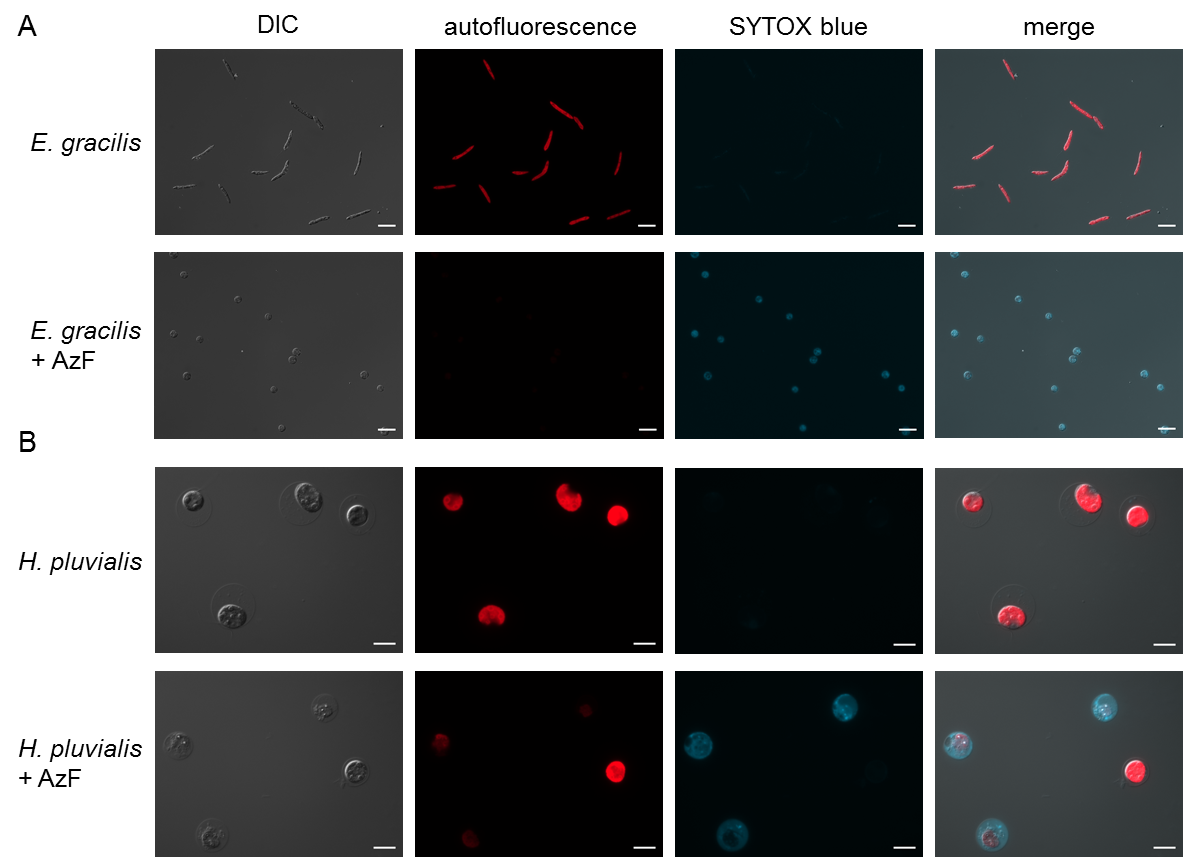
Cytoplasmic membrane permeabilization of algae from different phylogenetic backgrounds

Supplementary Figure 7: Effect of azalomycin F on the permeabilization of the cytoplasmic membrane. A. *E. gracilis* B. *H. pluvialis*. A positive autofluorescence and SYTOX Blue signal indicate live cells and cell death, respectively. 20 µg/ml AzF. Scale bars: *E. gracilis*, 50 µm; *H. pluvialis*: 20 µm.

- 1. Tripartite co-cultivation of *S. iranensis*, *A. nidulans* and *C. reinhardtii*


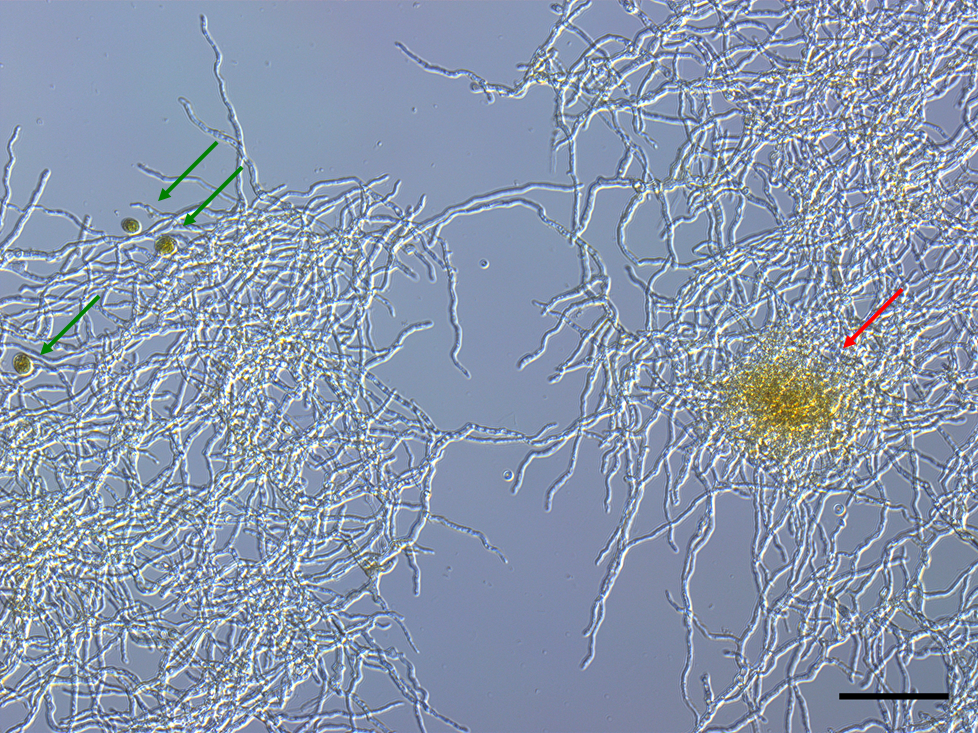


### Supplementary Figure 8: Co-cultivation of *S. iranensis*, *A. nidulans* and *C. reinhardtii*. All partners assemble within the fungal mycelium. Green arrows*: C. reinhardtii* cells. Red arrow: *S. iranensis* filaments. Scale bar: 50 µm.

- 1. Influence of azalomycin F on *C. reinhardtii* and various fungi


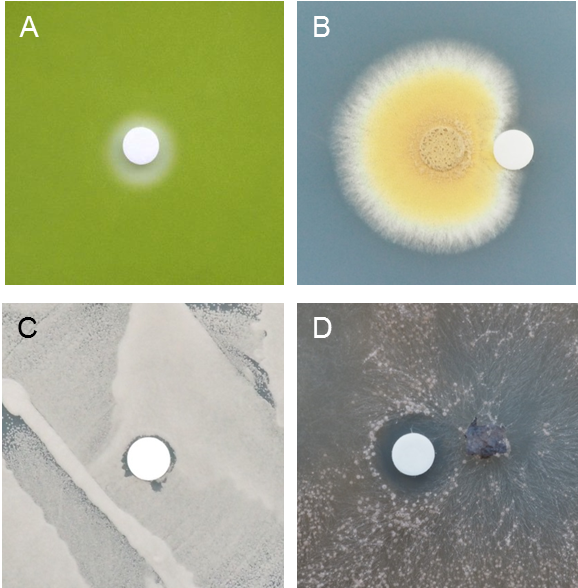


Supplementary Figure 9: *C. reinhardtii* and the tested fungi show variable sensitivity to azalomycin F. *C. reinhardtii* (A) is more sensitive to azalomycin F than *A. nidulans* (B) and *S. cerevisiae* (C). *S. macrospora* (D) is also susceptible to the applied amount of azalomycin F. The cotton disc was loaded with 12 µg azalomycin F and *A. nidulans* and *S. macrospora* were inoculated in 1 cm distance. *C. reinhardtii* was inoculated into the agar. *S. cerevisiae* was spread onto the agar.

- 1. Test of capability of *Saccharomyces cerevisiae* and *Sordaria macrospora* to attract and protect *C. reinhardtii*


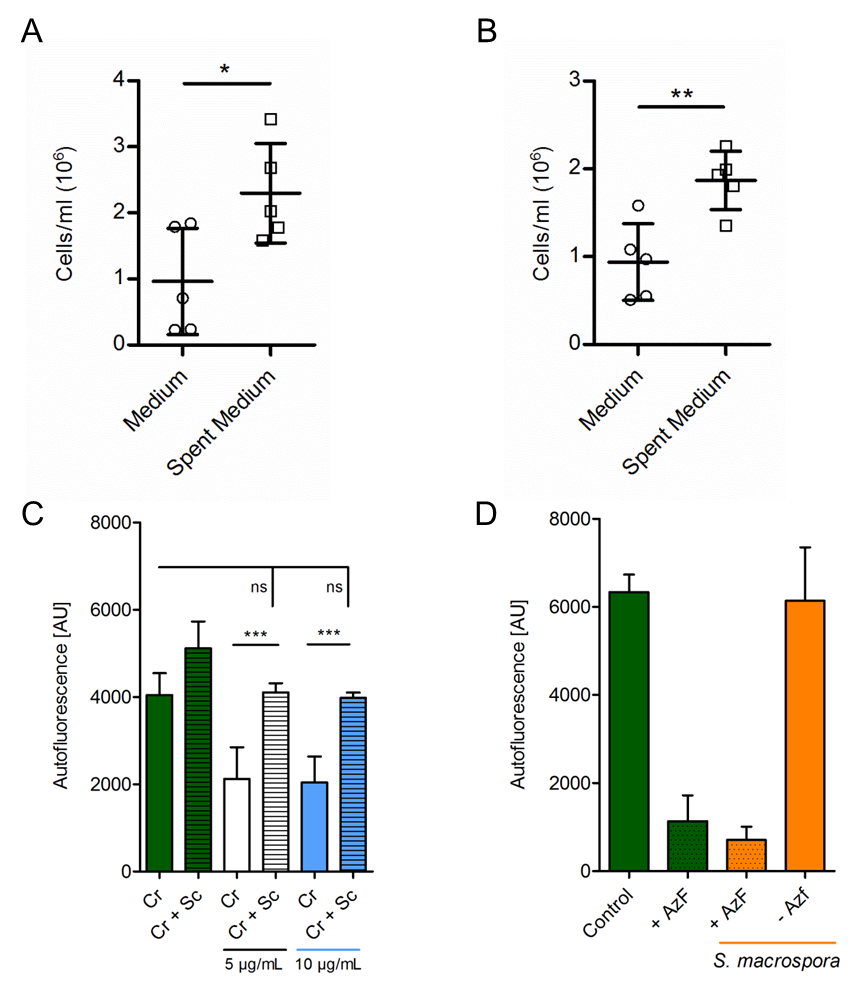


### Supplementary Figure 10: *S. cerevisiae* and *S. macrospora* are able to attract *C. reinhardtii*, but only *S. cerevisiae* can protect it from azalomycin F. A and B: Chemotaxis assay using supernatants of *S. cerevisiae* (A) and *S. macrospora* (B). C and D: Autofluorescence of *C. reinhardtii* (Cr) in co-culture with *S. cerevisiae* (Sc) treated with 0, 5 or 10 µg/ml azalomycin F respectively (C). D: Co-culture of *C. reinhardtii* and *S. macrospora* treated with 0 or 1 µg/ml azalomycin F. *** P ≤ 0.001; ns, not significant. Calculated from at least three biological replicates, error bars represent standard deviation

- 1. Association of *S. cerevisiae* and *S. macrospora* and *C. reinhardtii*


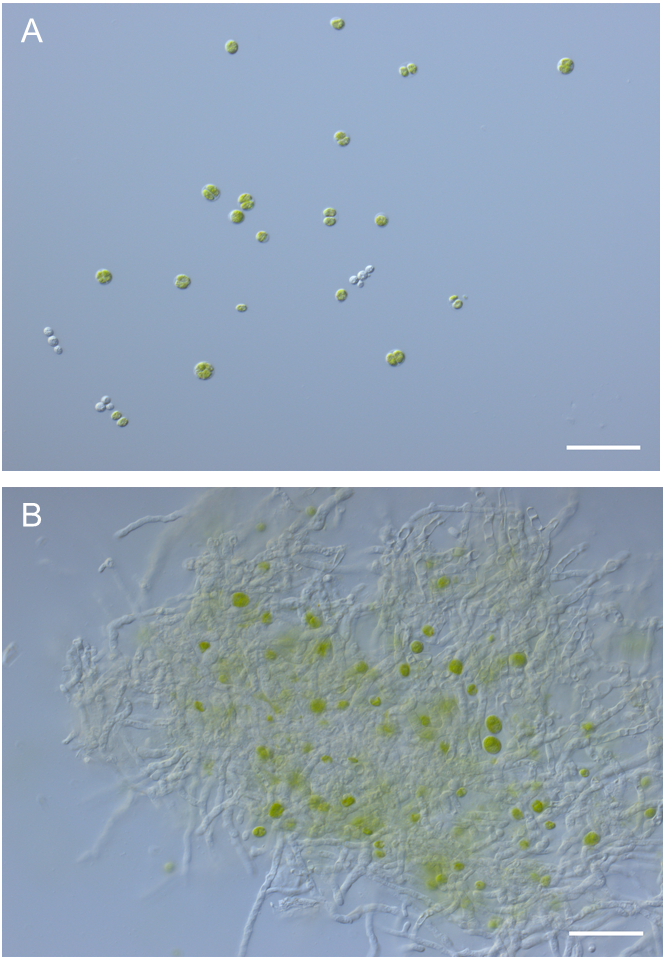


### Supplementary Figure 11: A: Co-cultivation of *C. reinhardtii* and *S. cerevisiae* does not lead to tight association of the partners (green: *C. reinhardtii*, colorless: *S. cerevisiae*). B: Co-cultivation of *C. reinhardtii* and *S. macrospora* leads to accumulation of algae in the fungal mycelium. Scale bars: 50 µm.

- 1. Supplementary data on non-protecting polar lipids and amphotericin B precomplexation


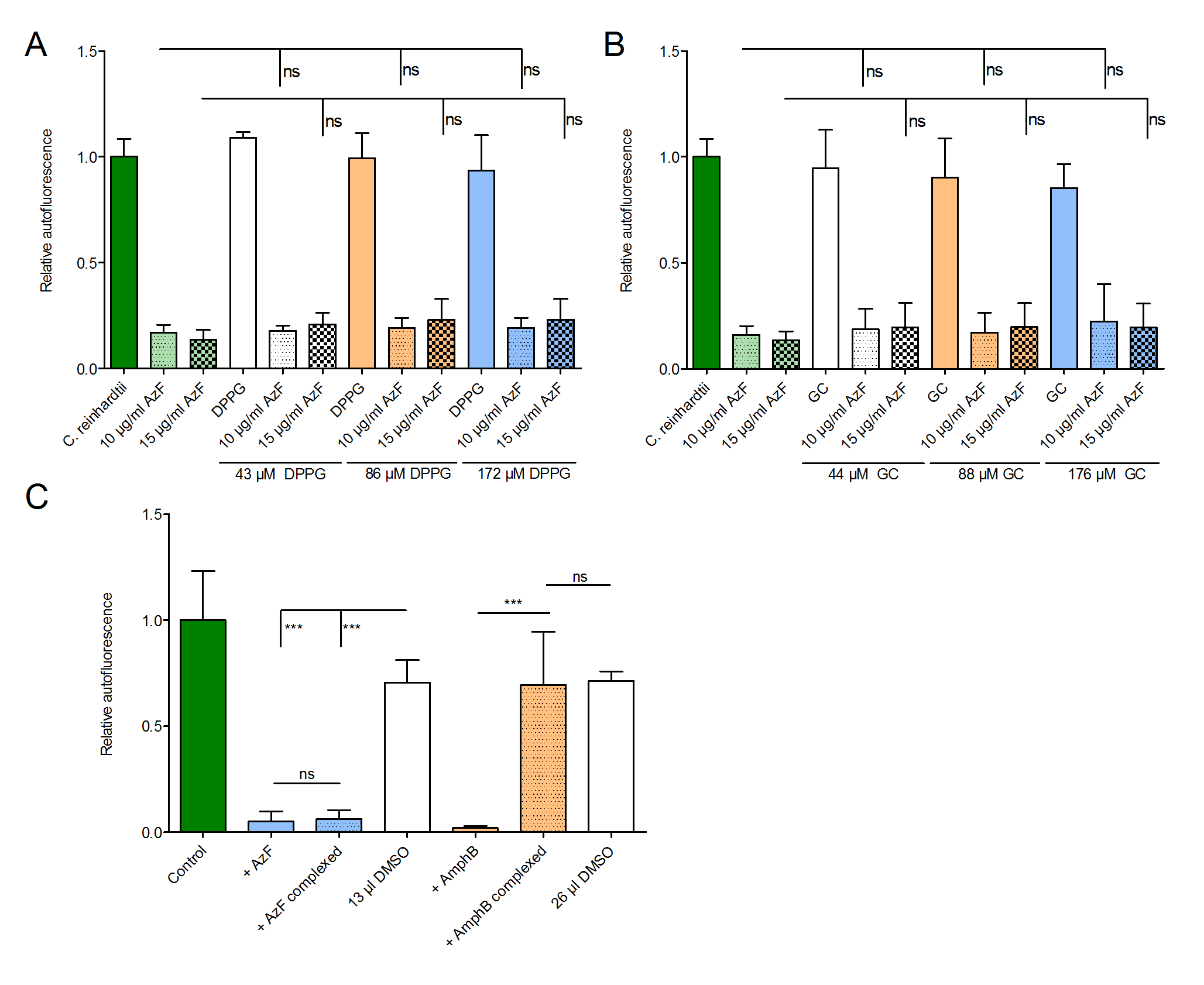


### Supplementary Figure 12: DPPG and GC did not protect *C. reinhardtii* against azalomycin F. A and B. Relative autofluorescence of *C. reinhardtii* treated with 0, 10 and 15 µg/ml azalomycin F and various concentrations of 1,2-dipalmitoyl-sn-glycero-3-phospho-(1'-rac-glycerol) (DPPG) and glucosylceramide (GC). C. Relative autofluorescence of *C. reinhardtii* treated with azalomycin F (AzF) or amphotericin B (AmphB, control) complexed with ergosterol and uncomplexed. Ergosterol-treated AzF showed no reduction of activity, while pre-complexed AmphB significantly lost activity. *P ≤ 0.05; **P ≤ 0.01; P *** P ≤ 0.001; ns, not significant. calculated from at least three biological replicates, error bars represent standard deviation.

- 1. qRT-PCR of genes involved in lipid biosynthesis of *A. nidulans*

**

**

Supplementary Figure 13: qRT-PCR analysis of genes involved in lipid biosynthesis. The expression of the genes was compared to the house-keeping gene gamma-actin (*AN6542*) and time-point 0 (t_0_) of the *A. nidulans* monoculture served as a reference (dashed line). t_5_ depicts the time point 5 hours after cultivation, which matches the time of co-cultivaton that was applied in the protection assay against azalomycin F. *AN0913* is involved in the biosynthesis of phosphatidylinositol, *AN4332* (*barA*) encodes a ceramide synthase, which drives the production of sphingolipids. *AN6530* encodes a protein of the glycerolipid biosynthesis pathway and *AN6580* is annotated as cardiolipin synthase.

References:

Anderson TM, Clay MC, Cioffi AG, Diaz KA, Hisao GS, Tuttle MD *et al.* (2014). Amphotericin forms an extramembranous and fungicidal sterol sponge. *Nature Chemical Biology* **10:** 400-406.

Bicudo CEM, Menezes M (2016). Phylogeny and Classification of Euglenophyceae: A Brief Review. *Frontiers in Ecology and Evolution* **4**.

Birch M, Drucker DB, Riba I, Gaskell SJ, Denning DW (1998). Polar lipids of *Aspergillus fumigatus, A. niger, A. nidulans, A. flavus* and *A. terreus*. *Medical Mycology* **36:** 127-134.

Brakhage AA, Van den Brulle J (1995). Use of reporter genes to identify recessive *trans*-acting mutations specifically involved in the regulation of *Aspergillus nidulans* penicillin biosynthesis genes. *J Bacteriol* **177:** 2781-2788.

Datsenko KA, Wanner BL (2000). One-step inactivation of chromosomal genes in *Escherichia coli* K-12 using PCR products. *Proceedings of the National Academy of Sciences of the United States of America* **97:** 6640-6645.

Esser K (1982). *Cryptogams: Cyanobacteria, Algae, Fungi, Lichens*: Cambridge University Press, London, United Kingdom.

Gorman D, Levine R (1965). Cytochrome f and plastocyanin: their sequence in the photosynthetic electron transport chain of *Chlamydomonas reinhardi.* *Proceedings of the National Academy of Sciences of the United States of America* **54:** 1665-1669.

Guiry DM, Guiry GM (2019). AlgaeBase. World-wide electronic publication, National University of Ireland, Galway: <http://www.algaebase.org>.

Gust B, Challis GL, Fowler K, Kieser T, Chater KF (2003). PCR-targeted *Streptomyces* gene replacement identifies a protein domain needed for biosynthesis of the sesquiterpene soil odor geosmin. *Proceedings of the National Academy of Sciences of the United States of America* **100:** 1541-1546.

Hamedi J, Mohammadipanah F, Klenk HP, Potter G, Schumann P, Sproer C *et al.* (2010). *Streptomyces iranensis* sp. nov., isolated from soil. *International Journal of Systematic and Evolutionary Microbiology* **60:** 1504-1509.

Hoffmann T, Dorrestein PC (2015). Homogeneous matrix deposition on dried agar for MALDI imaging mass spectrometry of microbial cultures. *Journal of the American Society for Mass Spectrometry* **26:** 1959-1962.

Hom EFY, Murray AW (2014). Niche engineering demonstrates a latent capacity for fungal-algal mutualism. *Science* **345:** 94-98.

Kobayashi M, Kakizono T, Nagai S (1991). Astaxanthin production by a green alga, *Haematococcus pluvialis* accompanied with morphological changes in acetate media. *Journal of Fermentation and Bioengineering* **71:** 335-339.

MacNeil DJ, Gewain KM, Ruby CL, Dezeny G, Gibbons PH, MacNeil T (1992). Analysis of *Streptomyces avermitilis* genes required for avermectin biosynthesis utilizing a novel integration vector. *Gene* **111:** 61-68.

Netzker T, Schroeckh V, Gregory MA, Flak M, Krespach MKC, Leadlay PF *et al.* (2016). An efficient method to generate gene deletion mutants of the rapamycin-producing bacterium *Streptomyces iranensis* HM 35. *Applied and Environmental Microbiololgy* **82:** 3481-3492.

Paget MS, Chamberlin L, Atrih A, Foster SJ, Buttner MJ (1999). Evidence that the extracytoplasmic function sigma factor sigmaE is required for normal cell wall structure in *Streptomyces coelicolor* A3(2). *J Bacteriol* **181:** 204-211.

Schroeckh V, Scherlach K, Nützmann HW, Shelest E, Schmidt-Heck W, Schuemann J *et al.* (2009). Intimate bacterial-fungal interaction triggers biosynthesis of archetypal polyketides in *Aspergillus nidulans*. *Proceedings of the National Academy of Sciences of the United States of America* **106:** 14558-14563.

Silva PC (1980). Names of classes and families of living algae: with special reference to their use in the index nominum genericorum (plantarum).

Stringer MA, Dean RA, Sewall TC, Timberlake WE (1991). Rodletless, a new *Aspergillus* developmental mutant induced by directed gene inactivation. *Genes Dev* **5:** 1161-1171.

Xu W, Zhai G, Liu Y, Li Y, Shi Y, Hong K *et al.* (2017). An iterative module in the azalomycin F polyketide synthase contains a switchable enoylreductase domain. *Angewandte Chemie International Edition* **56:** 5503-5506.
